# VIRMA modulates function of photoreceptor cells through m^6^A modification and alternative splicing

**DOI:** 10.1172/jci.insight.197880

**Published:** 2026-03-19

**Authors:** Wenjing Liu, Xiaojing Wu, Rong Zou, Fan Zhang, Yudi Fan, Kuanxiang Sun, Liping Yang, Jiang Hu, Lin Zhang, Xianjun Zhu

**Affiliations:** 1 Department of Orthopedics and; 2 The Sichuan Provincial Key Laboratory for Human Disease Gene Study and Center for Medical Genetics, Sichuan Provincial People’s Hospital, School of Medicine, University of Electronic Science and Technology of China, Chengdu, Sichuan, China.; 3 Beijing Chinagene Co. LTD, Beijing, China.; 4 Department of Ophthalmology, Third Hospital, Peking University, Beijing, China.; 5 Sichuan-Chongqing Joint Key Laboratory for Pathology and Laboratory Medicine, Jinfaeng Laboratory, Chongqing, China.

**Keywords:** Neuroscience, Ophthalmology, Epigenetics, Neurodegeneration, RNA processing

## Abstract

*N*^6^-methyladenosine (m^6^A) modification is the most prevalent posttranscriptional epigenetic modification in mammalian mRNAs, and it has been implicated in the regulation of nervous system development by modulating mRNA metabolism. VIRMA is the largest core subunit of the m^6^A methyltransferase complex and is essential for the assembly and stability of the m^6^A methyltransferase complex. In the retina, m^6^A methylation modification is widely distributed in various cellular layers and is essential for retinal homeostasis. Here, we demonstrate that VIRMA-mediated m6A modification is essential for retinal homeostasis. Loss of *Virma* in retinal rod cells resulted in abnormal reduction in m^6^A methylation levels, along with impaired photoreceptor function and degeneration. Mechanically, *Virma* depletion in photoreceptors dampened the m^6^A modification level of visual perception–associated genes, resulting compromised visual function and photoreceptors degeneration. Moreover, *Virma* interacted with splicing factor to regulate the alternative splicing events of retina function–related genes such as *Polg2*, which contributes to photoreceptor damage. Reintroduction of normal *Virma* expression colonially rescued photoreceptor degeneration. Collectively, our data elucidate the important role of *Virma*-mediated m^6^A modification in photoreceptor function and suggest that epigenetic modulation could serve as a potential target to treat these blinding diseases.

## Abstract 2

Restoring epigenetic regulator Virma prevents photoreceptor cell death in the retina, rescuing vision and offering a new strategy against blindness.

## Introduction

Two morphologically distinct types of photoreceptors in the retina, rod cells and cone cells, are central to visual perception, converting light stimuli into electrical signals that are transmitted to the brain for further processing (1). Inherited retinal dystrophies (IRDs) are a group of retina disorders characterized by photoreceptor death, leading to progressive vision loss (2–4). Retinitis pigmentosa (RP) is the most common IRD, characterized by primary degeneration of rod photoreceptors followed by the loss of cone photoreceptors, with a prevalence of approximately 1 in 4,000 individuals (5, 6). The currently identified RP-causing genes account for only about 70% of cases (7), suggesting that there are yet unknown genetic factors that can cause RP. This highlights the need for in-depth exploration of other regulator layers, including gene regulation and posttranscriptional modifications, in the pathogenesis of RP.

Epigenetic regulation has been identified to play a crucial role in the regulation of numerous physiological and pathophysiological processes (8, 9). *N*^6^-methyladenosine (m^6^A) is the most prevalent and conserved posttranscriptional epigenetic modification in eukaryotic RNAs, especially within higher eukaryotic RNAs (8, 10, 11). The dynamic m^6^A modification is installed by a methyltransferase complex (known as writers), including METTL3, METTL14, WTAP, HAKAI, VIRMA, METTL16, RBM15, and ZC3H13, and removed by demethylases (known as erasers), such as FTO and ALKBH5 (12–20). Specific RNA-binding proteins (known as readers), including YTHDF1/2/3, YTHDC1/2, IGF2BP1/2/3, and HNRNPC/A2B1, can selectively recognize the m^6^A modified mRNAs and, consequently, affect RNA export, stability, splicing, and translation (21–26). Genetic knockout and gene knockdown studies on m^6^A writers, erasers, and readers have shown that m^6^A plays important roles in regulating various aspects of organism development, physiology, and disease progression (27–29). In recent years, the role of m^6^A methylation in the development and homeostasis of retina has been well-established. For instance, *Mettl3* deficiency in retinal progenitor cells distorts late-stage retinogenesis (29). Conditional knockout of *Mettl14* in retinal photoreceptors causes impaired visual function and photoreceptor death (30). In addition, YTHDC1 directly binds to the lncRNA IPW (imprinted in Prader–Willi syndrome) to regulate retinal pigment epithelium (RPE) apical–basal polarization in patients with AMD (31). VIRMA is the largest known protein in the methyltransferase complex, and it is responsible for recruiting the catalytic core components METTL3/METTL14/WTAP to guide region-selective m^6^A methylation (32, 33). This underscores the pivotal role of VIRMA in the assembly of the m^6^A writer complex. In addition, VIRMA depletion led to the most obvious reduction in m^6^A levels compared with METTL3/14/WTAP knockdown cells, suggesting the central role of VIRMA in m^6^A modification (12, 34–36). Recently, VIRMA has been proven to play a key role in central nervous system diseases (37, 38). However, whether VIRMA-mediated m^6^A methylation, like other m^6^A modification components, also plays a crucial role in maintaining neuronal retina function remains unclear.

In addition, *VIRMA* has been reported to be involved in hepatocellular carcinoma, breast cancer, and mouse follicular development by regulating alternative splicing (AS) events (39–41). AS is a common mechanism for tissue-specific and development-specific regulation of gene expression in eukaryotes (42–44). Particularly high levels of AS are found in neuronal tissues, as these tissues maintain delicate cell subcellular diversity (45). Retina development relies on finely regulated cell type–specific gene expression, and AS plays a crucial role in contributing to this intricate development process (45, 46). It is no surprise that aberrant AS can impact retinal cell survival and function, resulting numerous IRDs, including RP, cone-rod dystrophy, and Usher syndrome (47). Whether VIRMA also plays a role in modulating AS events in photoreceptor remain unclear.

Here, we investigated the function of VIRMA in photoreceptors by generating retina-specific *Virma*-knockout mice using the *RHO-Cre* and *HRGP-Cre* lines. Our study found that loss of *Virma* in rods led to impaired visual function and degeneration of rods. Analogously, deficiency of *Virma* in cones caused death of cone cells. Further investigation revealed that *Virma* deficiency resulted in reduced global retina m^6^A methylation levels. Mechanistically, a comprehensive multi-omics analysis illustrated that decreased m^6^A methylation level via inactivation of VIRMA resulted in reduced expression level of multiple visual perception–associated genes. VIRMA also mediates AS events in photoreceptors, contributing to photoreceptor degeneration in RKO mice. Reintroduction of functional *Virma* expression in rods successfully rescued the photoreceptor degeneration. Therefore, our study revealed important mechanisms by which VIRMA regulates photoreceptor function and survival, highlighting key roles of VIRMA-mediated m^6^A modification and splicing in the retina.

## Results

### VIRMA deficiency in rod cells causes impaired visual function.

m^6^A modification has been reported to be involved in maintaining retinal homeostasis in several mouse models, as described in the Introduction. Although previous study illustrated that conditional knockout of the *Mettl3/14* or *Ythdc2* impaired photoreceptor function and survival in mice (30, 48, 49), whether other components involved in m^6^A modification could modulate photoreceptor function and survival remains unclear. To address this question, we focused on VIRMA, a structural component of the m^6^A methyltransferase complex, as its depletion leads to the biggest downregulation of m^6^A modification compared with other key members such as METTL3, METTL14, and WTAP (12). Immunofluorescence staining demonstrated that VIRMA was broadly expressed in nucleus of outer nuclear layer (ONL), inner nuclear layer, and ganglion cell layer in retina labeled with anti-VIRMA antibody, suggesting a potential functional role of VIRMA in retina (Supplemental Figure 1A; supplemental material available online with this article; https://doi.org/10.1172/jci.insight.197880DS1). To determine the function of VIRMA in photoreceptor cells, we crossed *Virma*-floxed mice with *RHO-Cre* (50) and *HRGP-Cre* transgenic mice (51), which express Cre specifically in the rod and cone cells, respectively (Supplemental Figure 1A). Genotyping confirmed the deletion of exon 2 in homozygotes, and the littermates of the progeny were distinguished as follows: *Virma^loxp/loxp^; RHO-Cre* (hereafter called RKO), *Virma^loxp/loxp^; HRGP-Cre* (hereafter called HKO), and *Virma^loxp/loxp^* (used as control [Ctrl]) mice (Supplemental Figure 1B). To confirm the cell type–specific expression of our Cre lines, we introduced *ROSA26*-tdTomato reporter mice to visualize the specific expression of *RHO-Cre* and *HRGP-Cre* in retina rod and cone photoreceptor cells, respectively. As expected, tdTomato fluorescence was detected in the ONL of *RHO-Cre* mice (Supplemental Figure 1, C and D). Given that rod cells constitute 97% in murine photoreceptors (52), the widespread tdTomato signal in the ONL of *RHO-Cre* mice supports its specificity for rod cells (Supplemental Figure 1D). As revealed by Western blot, RT-qPCR, and immunofluorescence staining, the expression level of *Virma* was drastically reduced in the retina, confirming the successful generation of *Virma* photoreceptor-specific knockout mice (Supplemental Figure 1, E and F). In *HRGP-Cre* mice, tdTomato fluorescence was also observed in the ONL (Supplemental Figure 1G). High-magnification costaining with the cone-specific marker cone arrestin (cArr) revealed precise colocalization of the tdTomato signal within cArr-positive cells, suggesting the precise excision.

**Figure 1:**
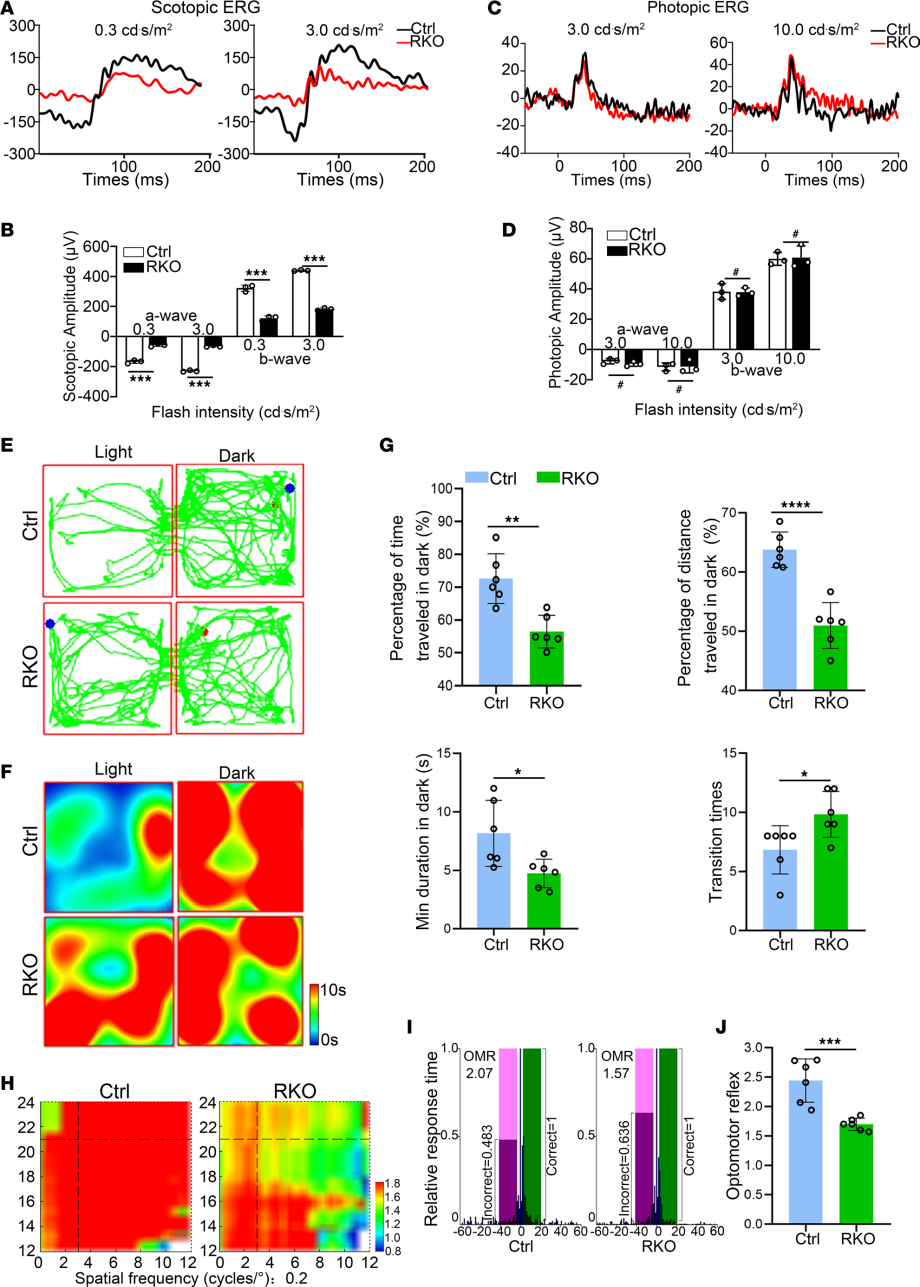
Impaired visual function in rod-specific *Virma*-depleted mice. (**A**) Scotopic ERGs were recorded with increasing light intensities from dark-adapted Ctrl and RKO mice at 4 weeks of age. (**B**) Statistical analysis for the amplitudes of the a-wave and b-wave under scotopic conditions (Student’s *t* test, *n* = 3). (**C**) Photopic ERGs were recorded from Ctrl and RKO mice at 4 weeks of age. (**D**) Statistical analysis for the amplitudes of the a-wave and b-wave under photopic conditions (Student’s *t* test, *n* = 3). (**E** and **F**) The representative traveling trajectories (**E**) and heatmap recordings for time spent in distinct regions (**F**) of the light-dark box at 2,000 lux luminance. (**G**) Statistical analysis based on behavioral parameters, including time, distance traveled, and duration as well as transition between the chambers (Student’s *t* test, *n* = 6). (**H**) Representative heatmaps of the visual stimuli–driven optomotor responses at 0.2 cycles/° spatial frequency. (**I**) Representative images of OMR values. The response time in either stimulus direction (positive value, light green window) or opposite direction (negative value, light magenta window) at a specific velocity threshold was normalized to the maximal response time, set as 1, and presented as a blue bar. (**J**) Statistical analysis of optomotor response (Student’s *t* test, *n* = 6). Data are presented as the mean ± SD. *P < 0.05; **P < 0.01; ***P < 0.001; ****P < 0.0001. A lack of significant difference is indicated by #.

To assess the visual function in *Virma* RKO mice, scotopic electroretinograms (ERGs) were performed on Ctrl and RKO mice at 4 weeks of age. Scotopic ERG responses in the RKO retinas exhibited diminished amplitudes for both a-waves and b-waves when compared with those of age-matched controls at each flash intensity (Figure 1, A and B). The average amplitudes of the a- and b-waves of RKO mice were reduced by approximately 69% and 62%, respectively. In contrast, photopic ERG traces, reflecting cone photoreceptor function, appeared normal in RKO mice at this time point (Figure 1, C and D). These results indicate that the rod photoreceptor function was significantly compromised by the loss of *Virma*. We then carried out behavioral assessments for visual function via the light-dark transition test and optomotor response test. The preferred movements in dark and light were intuitively presented in traveling trajectories (Figure 1E), and heatmaps documented the time spent at in different regions of the light-dark box (Figure 1F). RKO mice showed an apparent preference for staying in the dark chamber for over 55% of time at 2,000 lux luminance and traveled around 50% of distance in the dark, both of which were lower than either of those in Ctrl mice (Figure 1G). Meanwhile, the minimal duration time in dark was significant decreased, while the transition time increased (Figure 1G). In the C57BL/6J mouse strain, the ratio of the number of times of head movement responded in the correct and incorrect directions, namely optomotor reflex (OMR), is most significantly observed at a spatial frequency of 0.2 cycles/° (53). Therefore, a spatial frequency of 0.2 cycles/° was chosen to evaluate the effect of *Virma* deficiency on visual function in mice as previously described (53). The overall OMR values in Ctrl context exhibited equal or higher than 2.0, while in RKO mice, the OMR value was apparently reduced to 1.5 (Figure 1, H–J). These results indicate that the m^6^A writer VIRMA positively regulates rod photoreceptor function in vivo and *Virma* deletion compromise photoreceptor function.

### Rod cell degeneration in RKO mice.

**Figure 2:**
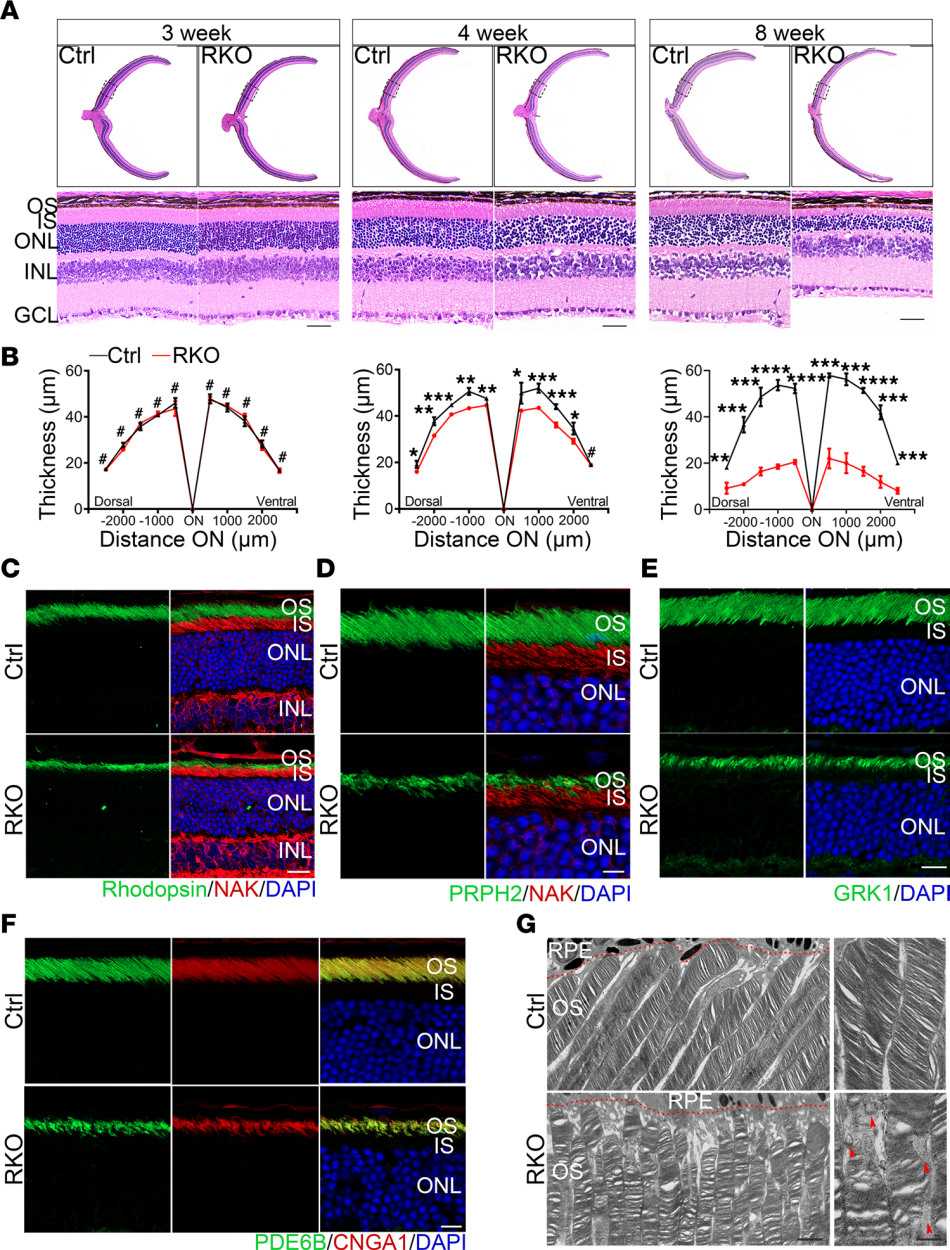
*Virma* deficiency leads to progressive rod cell loss in the retina. (**A**) H&E staining of paraffin sections of Ctrl and RKO retinas at the ages of 3 weeks, 4 weeks, and 8 weeks. Scale bars: 50 μm. (**B**) Quantitative analysis of the ONL thickness of the Ctrl and RKO retinas at defined ages (*n* = 3). Analyzed by multiple 2-tailed *t* tests with the Holm-Šídák method to correct for multiple comparisons. (**C**–**F**) Retinal cryosections from 4-week-old mice were stained with OS markers rhodopsin, PRPH2, GRK1, PDE6B, and CNGA1 and IS marker NaK ATPase. Scale bars: 25 μm. (**G**) Representative transmission electron microscopy images of photoreceptor outer segments in 4-week-old Ctrl and RKO mice. Red dashed line indicates the boundary between the RPE layer and the OS. Red arrowheads indicate the disorder of OS disk. RPE, retinal pigment epithelium; OS, outer segment; IS, inner segment; ONL, outer nuclear layer; INL, inner nuclear layer; GCL, ganglion cell layer. Data are presented as the mean ± SD. *P < 0.05; **P < 0.01; ***P < 0.001; ****P < 0.0001. A lack of significant difference is indicated by #.

To confirm morphological changes in mice, histological analysis was performed on the retinas of Ctrl and RKO mice at different ages. At 3 weeks of age, the cellular layers and retinal thickness in RKO mice were well maintained (Figure 2, A and B). In contrast, RKO mice exhibited gradual reduction in the outer segments (OS), inner segments (IS), and ONL of photoreceptors at 4 weeks, with these changes becoming more evident with age as a consequence of the *Virma* deletion (Figure 2, A and B). Intriguingly, the retinas of *Virma* RKO mice exhibited much more rapid degeneration compared with *Mettl3/14* RKO mice. The pathological phenotypes in the retinas of *Mettl3/14* RKO mice first appeared at 3 months and 3.5 months of age, respectively (Supplemental Figure 2), highlighting the key role of VIRMA in retina function.

The OS house a large amount of phototransduction proteins, all of which are synthesized in the IS and transported to the OS via the photoreceptor cilium. To elucidate the effect of *Virma* deficiency on the function of photoreceptor cells, we conducted immunofluorescence staining to assess the subcellular localization and expression of multiple key OS proteins in the RKO retina, including rhodopsin, PRPH2, GRK1, PDE6B, and CNGA1. In RKO retinas, these proteins retained their normal OS localization, but the OS appeared markedly shortened (Figure 2, C–F). This morphological phenotype may result from decreased protein synthesis or defects in cilium transport. To investigate this, we evaluated the morphology and structural changes of cilia using immunostaining and transmission electron microscopy (TEM). Cryosections of Ctrl and RKO animals were stained with centriole marker CEP164 and the ciliary marker acetylated α-tubulin. The results illustrated that, similar to those in Ctrl mice, the photoreceptor cilia in RKO mice appeared slender in their arrangement without noticeable length differences (Supplemental Figure 3, A–C). TEM results further corroborated these findings, showing that cilia in RKO mice also exhibited a typical 9+0 structure (Supplemental Figure 3D). As evidenced by TEM, a palisade pattern formed by the OS was observed on the RPE, displaying highly ordered OS disc in the 4-week-old Ctrl mice. However, age-matched RKO mice showed disorganized OS discs in photoreceptors cells, indicating the damage of the OS (Figure 2G).

In addition, the activation of resident retinal glial and immune cells is a key event in the progression of photoreceptor degeneration (54–56). We thus stained retinal sections from Ctrl and RKO mice for activated Müller and microglia markers. The abnormal migratory behavior stained with glial fibrillary acidic protein (GFAP) provides obvious evidence for Müller cell proliferation in RKO mice in response to retinal injury or stress (Supplemental Figure 4A). The ionized calcium-binding adapter molecule 1–immunoreactive (IBA1-immunoreactive) microglial cells had features typical of the resting state in Ctrl mice. In contrast, the IBA1-immunoreactive cells in RKO mice transformed into an amoeboid shape and infiltrated the ONL and both the inner and outer segments of photoreceptors (Supplemental Figure 4B). Western blot analysis further confirmed these results, as revealed by increased GFAP and IBA1 protein level in the RKO retinas (Supplemental Figure 4C). TUNEL staining suggested that gradual loss of photoreceptors in RKO mice primarily occurred via apoptosis (Supplemental Figure 4D).

Owing to the loss of trophic factors and structural support provided by neighboring rod photoreceptor cells, the degeneration of rods frequently leads to secondary morphological and physiological changes of cones, ultimately resulting in cell death (5, 57). While rods die first at 4 weeks, with ONL shrinking over time, cone structure begins to deteriorate at 8 weeks of age in RKO mice (Supplemental Figure 5, A–G). From 8 to 15 weeks of age in RKO mice, cones exhibited pronounced morphological alterations, mislocalization of cone OS proteins, and gradual cell death as a consequence of the progressive rod cell loss (Supplemental Figure 5, E–I). These results indicated that specific deletion of VIRMA in retina rod photoreceptor cells contributed to progressive photoreceptor cell loss.

### Cone cell defects in HKO mice.

**Figure 3:**
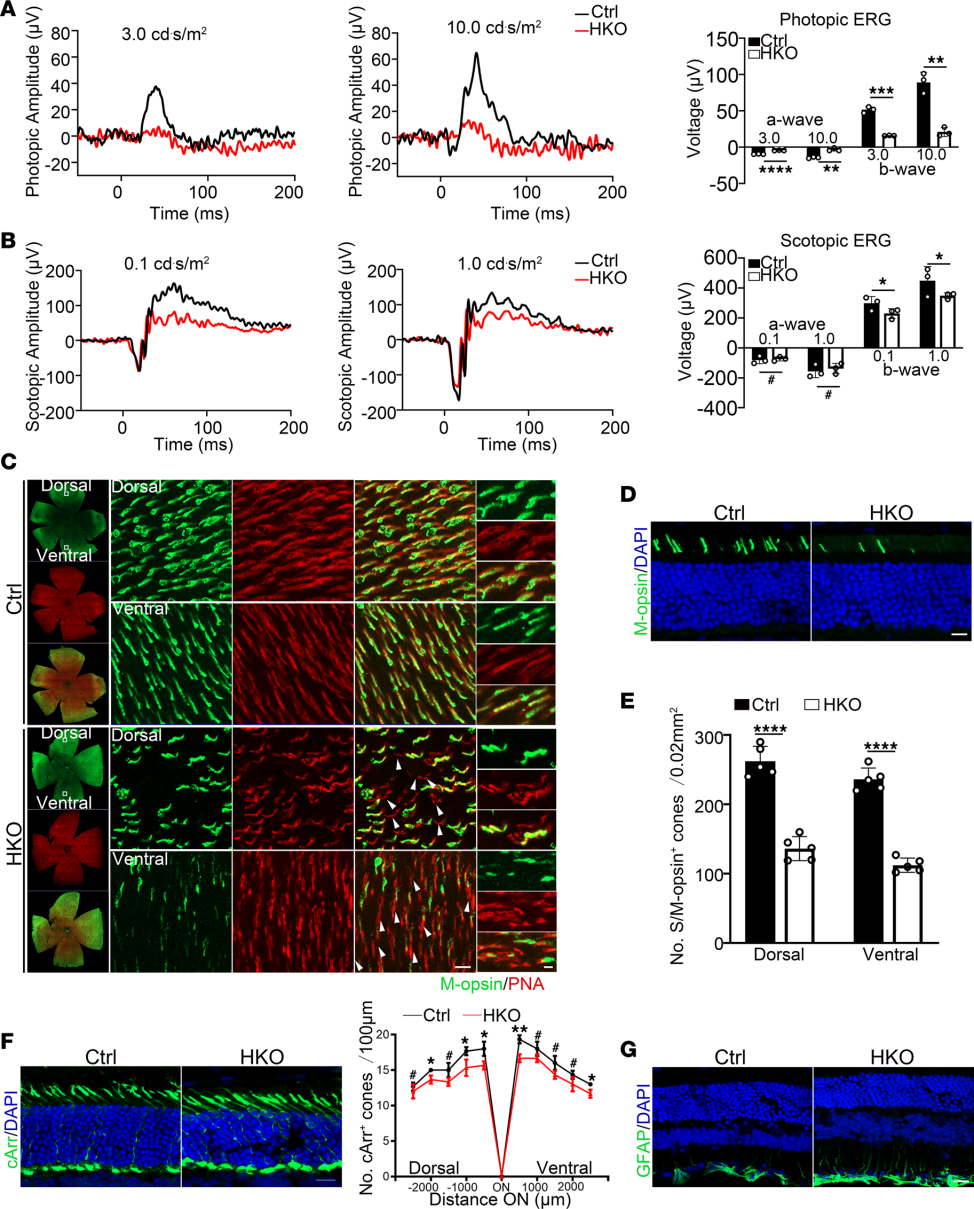
*Virma* depletion in cone cells leads to cone defects in the retina. (**A**) Representative photopic ERG traces and statistical analysis at identified flash intensities in mice at 8 weeks of age (Student’s *t* test, *n* = 3). (**B**) Representative scotopic ERG traces and statistical analysis at identified flash intensities in mice at 8 weeks of age (Student’s *t* test, *n* = 3). (**C**) Immunostaining of retina flat mount from 8-week-old Ctrl and HKO mice with M-opsin and PNA (red). Scale bars: 50 μm. Representative images from the dorsal and ventral retinal quadrant are shown. Scale bars: 20 μm. Inset images showed a cropped and zoomed image. Scale bars: 5 μm. White arrowheads indicate the lost and misshaped M-opsin–positive cones. (**D**) Retinal cryosections from 8-week-old Ctrl and HKO mice were labeled with M-opsin. Scale bars: 20 μm. (**E**) Quantification of the number of M-opsin–positive cones at both dorsal and ventral side of the retina per field (Student’s *t* test, *n* = 5). (**F**) Representative immunofluorescence images of cArr (green) of retina sections from 8-week-old Ctrl and HKO mice, and the number of cArr-positive cones per 500 μm field was quantified. Scale bars: 25 μm. Analyzed by multiple 2-tailed *t* tests with the Holm-Šídák method to correct for multiple comparisons. (**G**) Retinal sections from 8-week-old mice were stained with GFAP (green). Scale bars: 20 μm. Data are presented as the mean ± SD. *P < 0.05; **P < 0.01; ***P < 0.001; ****P < 0.0001. A lack of significant difference is indicated by #.

Rod cells detect light intensity, contributing to night and peripheral vision, while cone cells perceive color, supporting daylight and central vision (58). To further examine the role of VIRMA in photoreceptor function, we specifically knocked out *Virma* in cone cells using *HRGP-Cre*. Photopic ERG showed decreased a- and b-wave amplitudes in 8-week-old HKO mice compared with controls (Figure 3A). At this time point, scotopic a-wave amplitude was unaffected (Figure 3B). At 8 weeks of age, HKO mice showed marked and clear changes in cones morphology, along with a slight decrease in cone density (Figure 3, C–F). In the both dorsal and ventral retinas of HKO mice, the M-opsin–positive cones exhibited a significant decrease in number, accompanied by morphological disorganization and misshapen appearance (Figure 3, C–E). In contrast, the number of S-opsin–positive cones showed no significant change, with only minor morphological distortions (Supplemental Figure 6). Subsequently, starting from week 16, a sharp decline in the number of cones was observed in HKO mice, which became more evident with increasing age (Supplemental Figure 7). Degeneration of cones also triggered glial cell activation in the retina (Figure 3G). Misshaped and reduced cone cells observed in HKO mice suggested that *Virma* depletion resulted in the degeneration of cone cells.

### VIRMA depletion decreases the abundance of m^6^A methylation in the RKO retina.

**Figure 4:**
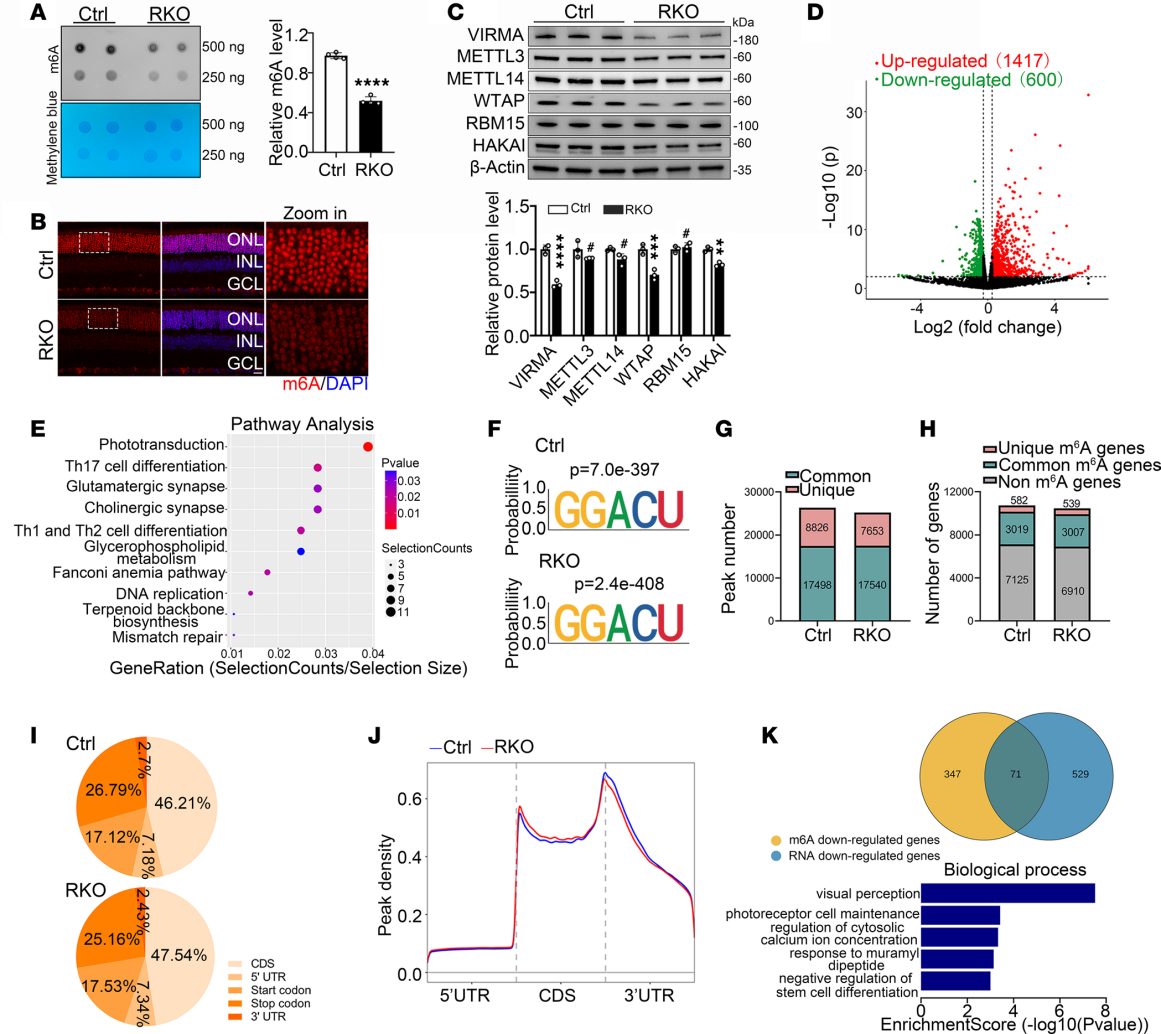
Loss of *Virma* led to reduction of global m^6^A modification abundance in the retina. (**A**) m^6^A dot blot assay of global m^6^A abundance in retinas from 4-week-old Ctrl and RKO mice using 250 ng or 500 ng mRNAs. Methylene blue staining was used as a loading control (Student’s *t* test, *n* = 4). (**B**) Immunofluorescence staining of m^6^A (red) of retina cryosections from 4-week-old mice. Scale bars: 20 μm; 5 μm (higher-magnification images). (**C**) Western blot and quantification of expression of m^6^A writers in 4-week-old mouse retinas (Student’s *t* test, *n* = 3). (**D**) Volcano plots of the significantly differentially expressed genes between Ctrl and RKO (fold change ≥1.2 and *P* < 0.05). (**E**) Pathway analysis in downregulated genes after *Virma* deletion. (**F**) Top consensus m^6^A motif identified by HOMER in Ctrl and RKO retinas. (**G**) Number of m^6^A peaks identified from MeRIP-seq in Ctrl and RKO retinas. (**H**) Number of m^6^A-modified genes identified from MeRIP-seq in Ctrl and RKO retinas. (**I**) Graphs illustrating the proportion distribution of the m^6^A peaks in mRNA transcripts from Ctrl and RKO retinas. (**J**) Distribution of m^6^A peaks across 5′ UTR, CDS, and 3′ UTR of mRNA in Ctrl and RKO retinas. CDS, code sequence. (**K**) A Venn diagram showing the shared genes with reduced m^6^A modification levels and mRNA expression levels, along with enriched GO terms for these 71 selected genes.

VIRMA serves as an interaction platform for the assembly of MACOM complex (including VIRMA, WTAP, HAKAI, RBM15, and ZC3H13), which is required for mRNA m^6^A methylation (18). We thus assessed the global profiles of m^6^A methylation levels in retina via dot blot assay, and the result revealed a 50% reduction of overall m^6^A levels in the RKO mice compared with the Ctrl mice (Figure 4A). Immunofluorescence staining further confirmed the aberrant downregulation of m^6^A levels in ONL of RKO mice (Figure 4B). We then explored whether the loss of VIRMA affects the expression and subcellular distribution of other MACOM members. To this end, we detect the protein levels of these members, and the data showed that only WTAP expression was downregulated upon VIRMA deletion, while the expression of other members remained unaffected, which consistent with findings from a previous report (32) (Figure 4C).

Previous studies suggest that m^6^A methyltransferases are predominately localized in the nuclear speckles, where m^6^A methylation occurs (15–17, 34). MACOM complex showed no obvious change in cellular localization upon VIRMA depletion (Supplemental Figure 8). These results suggest that VIRMA is not required for the nuclear localization of the MACOM complex, except for its effect on WTAP stabilization within the nucleus.

To investigate whether the functional defects in photoreceptor cells of RKO mice were attributable to decreased m^6^A modification and to elucidate the underlying molecular mechanisms, we comprehensively analyzed the retinal mRNA m^6^A modification landscape via methylated RNA immunoprecipitation–qPCR (MeRIP-qPCR) and the corresponding gene expression profiles via RNA-seq. The data revealed 600 downregulated genes and 1,417 upregulated genes with significant differences (fold change >1.2, *P* < 0.05) in transcript levels between Ctrl and RKO mice, and these genes were primarily enriched in the phototransduction signaling pathway (Figure 4, D and E). The m^6^A “GGACU” consensus motif was highly enriched in both Ctrl and RKO groups using the HOMER program (59, 60) (Figure 4F). In total, MeRIP-seq identified 26,324 m^6^A peaks from 3,601 m^6^A-modified transcripts in Ctrl retinas and 25,193 m^6^A peaks from 3,546 m^6^A-modified transcripts in RKO retinas. Following *Virma* deletion in rods, 539 genes gained m^6^A modification , 582 genes lost m^6^A modification, and 3,007 genes were common to both groups (Figure 4, G and H). Peak distribution analysis showed a similar pattern, with m^6^A sites enriched in both CDS and 3′ UTRs. The highest enrichment of m^6^A residues was located near the stop codon and 3′ UTRs (Figure 4, I and J), which is consistent with results from previous studies (32, 41). As is typical, less enrichment of m^6^A peaks appeared at 3′ UTR and near the stop codon in RKO retinas (Figure 4J). By intersecting the 600 downregulated genes identified from RNA-seq and 418 m^6^A-downregulated genes identified from MeRIP-seq, 71 candidate genes were selected (Figure 4K). Gene ontology (GO) analysis of these genes yielded the enrichment signaling pathways of biological process, and notable gene sets include visual perception and photoreceptor cell maintenance (Figure 4K). Therefore, we focused primarily on the alterations of these two signaling cascades in the subsequent investigation.

### Virma regulates the expression of visual perception–related genes through m^6^A modification.

**Figure 5:**
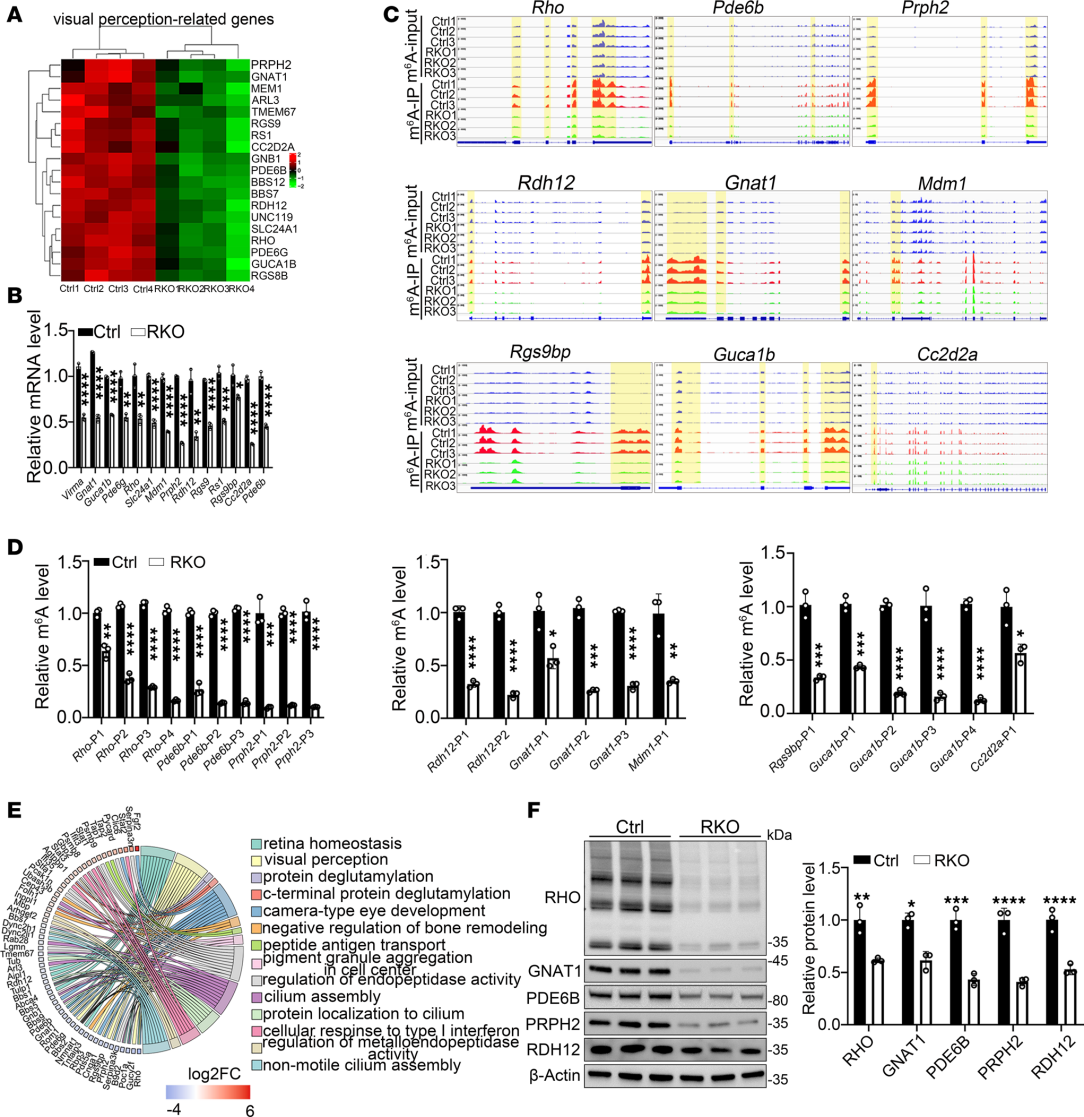
*Virma* regulated visual perception–associated gene expression via m^6^A modification in rods. (**A**) Heatmap of several visual perception–related genes identified in Figure 4. (**B**) RT-qPCR verified the mRNA expression for the indicated genes (Student’s *t* test, *n* = 3). (**C**) Integrative Genomics Viewer tracks of MeRIP-seq reads along indicated mRNAs. Normalized reads density levels are shown as blue (input), red (Ctrl), and green (RKO) shades, respectively. Three replicates are shown. (**D**) MeRIP-qPCR assay indicating the m^6^A modification levels for the indicated genes (Student’s *t* test, *n* = 3). (**E**) Chord plot of differentially expressed proteins in the RKO group compared with Ctrl groups. (**F**) Western blot and quantification of visual perception–related protein expression in retinas from 4-week-old mice. β-Actin served as control. Data are presented as the mean ± SD. *P < 0.05; **P < 0.01; ***P < 0.001; ****P < 0.0001.

Heatmaps of the visual perception–related genes showed significant downregulation at the mRNA level (Figure 5A), which was further validated by RT-qPCR showing reduced expression of *Gnat1*, *Guca1b*, *Prph2*, *Pde6b*, *Pde6g*, *Rgs9bp*, *Rs1*, *Prph2*, *Slc24a1*, *Rho*, *Mdm1*, *Cc2d2a*, and *Rgs9* in the retinas of RKO mice (Figure 5B). These genes also exhibited significant loss of m^6^A peak signals in response to *Virma* knockout, as confirmed by MeRIP-qPCR (Figure 5, C and D). Compared with the previously reported *Mett13/14* RKO mice, a greater number of visual perception–related genes were downregulated in the retinas of *Virma* RKO mice (30, 49). To further explore the mechanism underlying VIRMA’s regulation of photoreceptor function, we used a proteomic approach on retinas from Ctrl and RKO mice. GO enrichment analysis identified substantially altered categories of proteins following *Virma* deletion. The differently expressed proteins were mainly involved in retina homeostasis, visual perception, and protein deglutamylation (Figure 5E), which was closely aligned with the RNA-seq data. Western blot analysis also revealed a reduction in RHO, GNAT1, PDE6B, PRPH2, and RDH12 (Figure 5F). To determine whether m^6^A hypomethylation directly caused the downregulation of visual perception–related genes, we analyzed global m^6^A levels at postnatal week 3, prior to the onset of photoreceptor degeneration. Dot blot analysis revealed a significant reduction in overall m^6^A levels in the RKO mice compared with the Ctrl mice (Supplemental Figure 9A). At this early stage, H&E staining and immunostaining for key photoreceptor function or structural proteins showed no obvious difference between RKO and Ctrl retina sections (Figure 2A and Supplemental Figure 9, B–D). GFAP and TUNEL staining also showed no cellular stress or apoptosis in RKO photoreceptors (Supplemental Figure 9, E and F). Furthermore, RT-qPCR analysis revealed no significant difference in the expression of visual perception–related genes between RKO and Ctrl mice (Supplemental Figure 9G). Thus, m^6^A methylation reduction precedes both transcriptional decline and phenotypic changes, confirming its primary role in disease initiation. Collectively, these findings shed light on the epigenetic underpinnings that drive the progress of photoreceptor degeneration, with a marked decrease in m^6^A methylation level of visual perception–associated key molecules.

### Virma modulates the AS of Imphd1 and Polg2.

**Figure 6:**
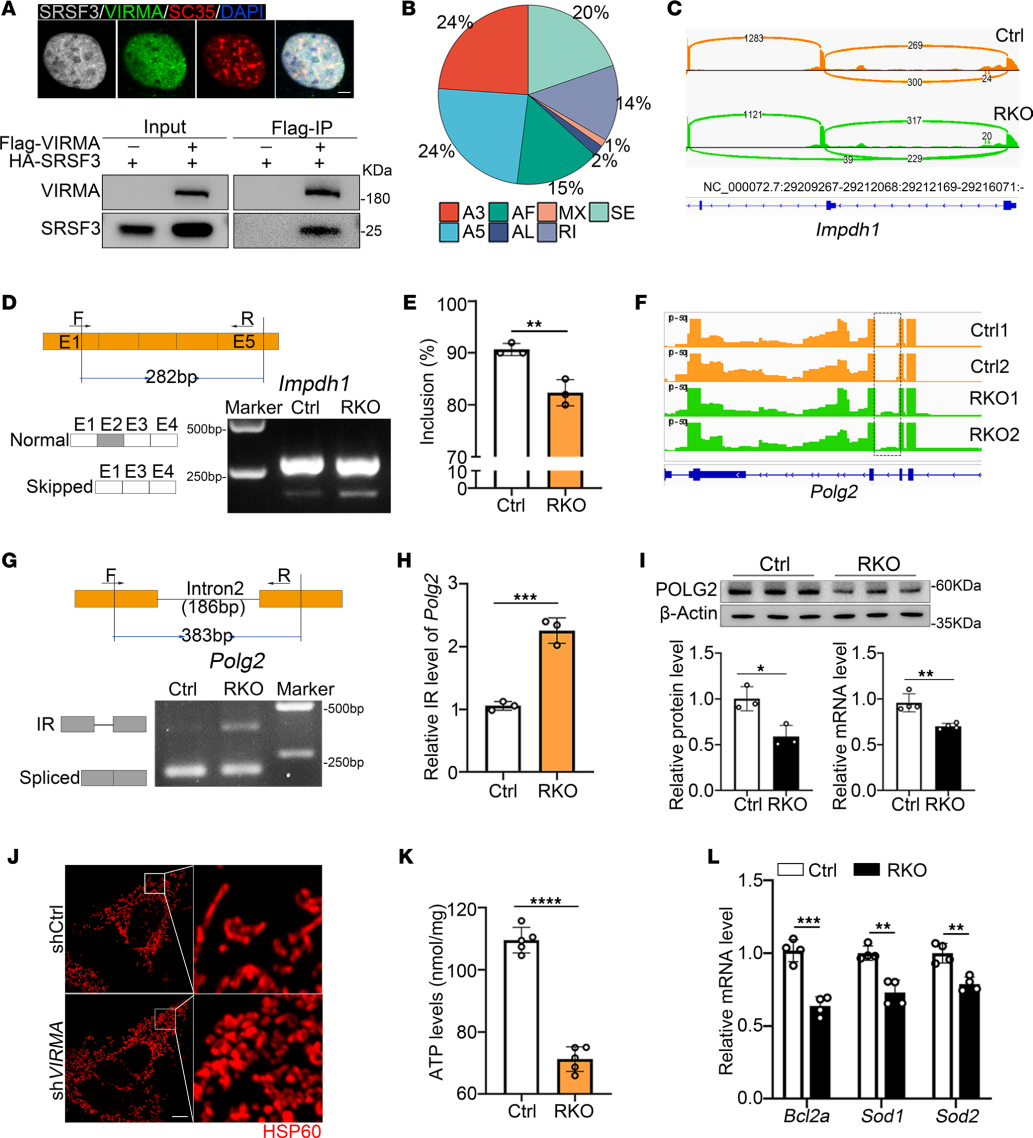
*Virma* is involved in alternative splicing of transcripts in retina. (**A**) Colocalization and coimmunoprecipitation analysis of VIRMA and SRSF3. Scale bar: 10 μm. (**B**) Seven types of alternative splicing events were identified in RKO retains. A3, alternative 3′ splice site; A5, alternative 5′ splice site; MX, mutually exclusive exon; SE, skipped exon; RI, retained intron; AF, alternative first exon; AL, alternative last exon. (**C**) Sashimi plot of *Impdh1* gene in Ctrl and RKO retinas. (**D** and **E**) RT-PCR validation of exon 2 skipping of *Impdh1* gene (Student’s *t* test, *n* = 3). (**F**) Wiggle plots showing intron retention of *Polg2*. (**G** and **H**) RT-PCR validation of intron retention of *Polg2* gene in RKO retinas (Student’s *t* test, *n* = 3). (**I**) Western blot and RT-qPCR analysis of POLG2 expression in Ctrl and RKO mice (Student’s *t* test, *n* = 3). (**J**) HSP60 (a mitochondrial marker) immunofluorescence staining of shCtrl and sh*VIRMA* 293STF cells. Scale bars: 20 μm; 5 μm (higher-magnification images). (**K**) ATP levels in fresh retains from 4-week-old Ctrl and RKO mice were measured using luciferase assay (Student’s *t* test, *n* = 5). (**L**) RT-qPCR analysis of the expression of mitochondrial function–related genes (Student’s *t* test, *n* = 4). Data are presented as the mean ± SD. *P < 0.05; **P < 0.01; ***P < 0.001; ****P < 0.0001.

VIRMA, like other known nuclear enzymes related to m^6^A modification, is localized to the nuclear speckles that enrich pre-mRNA processing factors (12, 16, 17, 34, 61) (Supplemental Figure 8). SRSF3, a member of serine/arginine rich protein family, is involved in regulating AS (62). It has been reported that the m^6^A-binding protein YTHDC1 can recruit SRSF3 to modulate the AS of target genes (63). Immunofluorescence showed that VIRMA colocalized with SRSF3 in the nuclear speckles, while coimmunoprecipitation confirmed the interaction between VIRMA and SRSF3 (Figure 6A), suggesting that VIRMA may also play a role in regulating AS. To confirm this, high-throughput transcriptomic was applied to measure the AS events in mRNAs potentially affected by VIRMA depletion in rods. A total of 452 significant AS events were identified (*P* < 0.05) in 205 genes using SUPPA2 (64), including 109 in alternative 5’ splice sites (A5SS), 108 in alternative 3’ splice sites (A3SS), 89 in skipped exons (SE), 69 in alternative first exon (AF), 62 in retained introns (RI), 9 in alternative last exon (AL), and 6 in mutually exclusive exons (MXE) under VIRMA deficiency (Figure 6B). Among them, 29 events were directly related to retina functions, and 19 gene were most attractive, as they have been shown to regulate nucleotides metabolism, visual phototransduction, ciliogenesis, mitochondrial dysfunction, and ubiquitination modification in retina (Supplemental Table 1). The results revealed that significant AS events in these genes, in response to VIRMA deletion, tended to promote exon skipping and intron retention (Supplemental Table 1). We next validated the reliability of high-throughput transcriptomic data by RT-PCR analysis of key retina function–related genes in Ctrl and RKO retinas: *Impdh1* (associated with purine metabolism), *Polg2* (mitochondrial DNA [mtDNA] replication related), and *Actg1* (engaged in synaptic function). Compare with controls, RKO mice exhibited increased skipping of exon 2 in *Impdh1* (Figure 6, C–E) and increased retention of intron 2 in *Polg2* (Figure 6, F–H). Retinal inosine monophosphate dehydrogenase 1(*IMPDH1*) encodes enzyme IMP dehydrogenase type 1, an enzyme that catalyzes the rate-limiting step of de novo guanine synthesis by converting inosine monophosphate (IMP) to xanthosine monophosphate (XMP) with the reduction of NAD (65). Mutations in *IMPDH1* were previously reported in patients with autosomal dominant RP 10 (66, 67), highlighting the critical role of this enzyme in maintaining photoreceptor function.

Intron retention level is negatively correlated with the steady-state expression of genes (68, 69), and RT-qPCR and Western blot further confirmed reduced expression of *Polg2* at both the mRNA and protein levels, respectively (Figure 6I). *Polg2*, encoding the α subunit of mtDNA polγ, has been identified to be associated with progressive external ophthalmoplegia accompanied by vision and hearing loss (70). Mitochondrial dysfunction is implicated in multiple common pathologies, including neurodegeneration, metabolic syndrome, and cancer (71–73). mtDNA plays a crucial role in mitochondria function, and any alterations in mtDNA replication can effect the normal function of mitochondria. Therefore, we first assessed the status of mitochondrial in the absence of *VIRMA* by immunostaining with heat shock protein (HSP60, a mitochondrial chaperonin) and found abnormal mitochondrial morphology following *VIRMA* deletion (Figure 6J). We also found that *Virma* loss decreased ATP production in RKO retinas (Figure 6K). *Bcl2a*, an antiapoptotic factor in the mitochondrial death pathway, showed decreased expression in RKO retina. Additionally, the mitochondrial detoxification enzymes *Sod1* and *Sod2* were downregulated in RKO retina as well (Figure 6L). These data suggested the presence of mitochondrial damage in RKO mice. In summary, the m^6^A methyltransferase VIRMA participates in posttranscriptional regulation by regulating AS in rod cells.

### Srsf3 depletion in retina rods causes impaired visual function.

**Figure 7:**
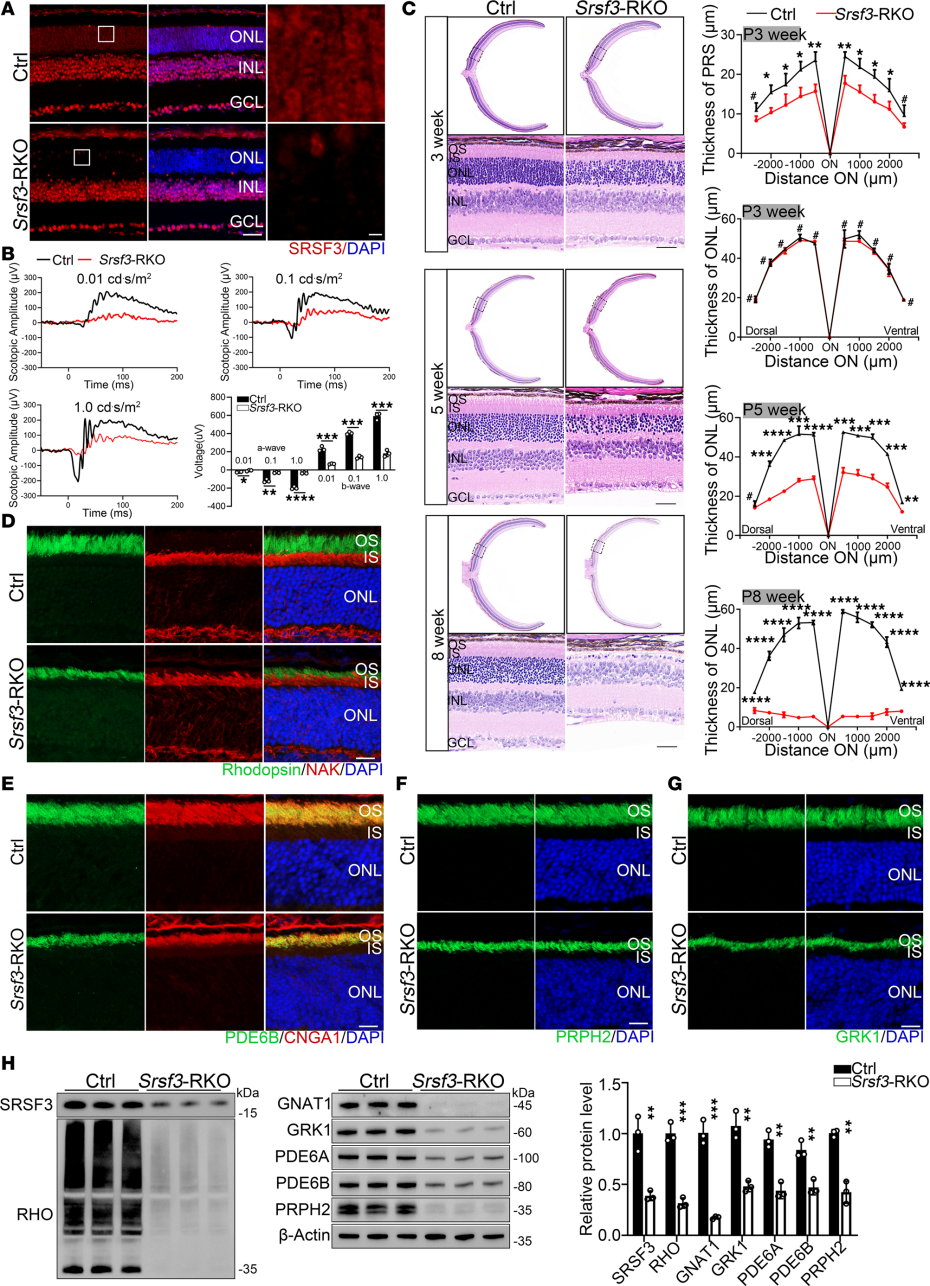
*SRSF3*-mediated alternative splicing is crucial for retina function. (**A**) Immunofluorescence staining of SRSF3 in Ctrl and *Srsf3*-RKO mice. Scale bars: 20 μm; 5 μm (higher-magnification images). (**B**) Representative scotopic ERG traces and statistical analysis at identified flash intensities in mice at 3 weeks of age (Student’s *t* test, *n* = 3). (**C**) H&E staining was performed on paraffin sections from *Srsf3*-RKO and Ctrl mice at 4, 5, and 8 weeks. The thickness of the photoreceptor segments (PRS) and ONL was quantified at defined ages (*n* = 3). Scale bar: 25 μm. Analyzed by multiple 2-tailed *t* tests with the Holm-Šídák method to correct for multiple comparisons. (**D**–**G**) Retinal cryosections from 3-week-old mice were stained with OS markers rhodopsin, PRPH2, GRK1, PDE6B, and CNGA1 and IS marker NaK ATPase. Scale bars: 25 μm. (**H**) Western blot and quantification analysis of OS key functional proteins (Student’s *t* test, *n* = 3). OS, outer segment; IS, inner segment; ONL, outer nuclear layer; INL, inner nuclear layer; GCL, ganglion cell layer, RPS, photoreceptor segment. Data are presented as the mean ± SD. *P < 0.05; **P < 0.01; ***P < 0.001; ****P < 0.0001. A lack of significant difference is indicated by #.

Retinal tissue exhibits one of the highest levels of AS, and AS makes a major contribution to gene expression during retinal development and homeostasis. Mutations in general splicing factors have been reported to be associated with RP in humans (74–76). Previous studies on AS focused on the spatial and temporal patterns of protein isoforms during retina development (77). Here, we generated rod cell–specific *Srsf3* knockout mice (*Srsf3*-RKO) to study the AS dysregulation in photoreceptor function. We first confirmed a greater reduction in SRSF3 expression in the retinas of *Srsf3*-RKO mice compared with Ctrl mice using immunofluorescence staining (Figure 7A). Scotopic ERG recordings revealed reduced amplitudes of both a- and b-waves in *Srsf3*-RKO mice compared with controls at each flash intensity at 3 weeks of age (Figure 7B). H&E staining showed a shorter photoreceptor segment in the *Srsf3*-RKO mice, with obvious photoreceptor loss at 3 weeks of age. However, *Srsf3*-RKO mice underwent rapid pathological changes: by 5 weeks of age, the ONL was reduced to approximately 50%, and by 8 weeks of age, the ONL was nearly completely lost (Figure 7C). Immunofluorescence staining (Figure 7, D–G) and Western blot (Figure 7H) further revealed reduced expression of OS structural and functional proteins in *Srsf3*-RKO retinas at 3 weeks of age, including rhodopsin, PDE6B, CNGA1, PRPH2, and GRK1. These data highlight the importance of a stable splicing process in maintaining retinal photoreceptor function.

### Rescue effect of Virma in rod cells on photoreceptor degeneration in RKO mice.

**Figure 8:**
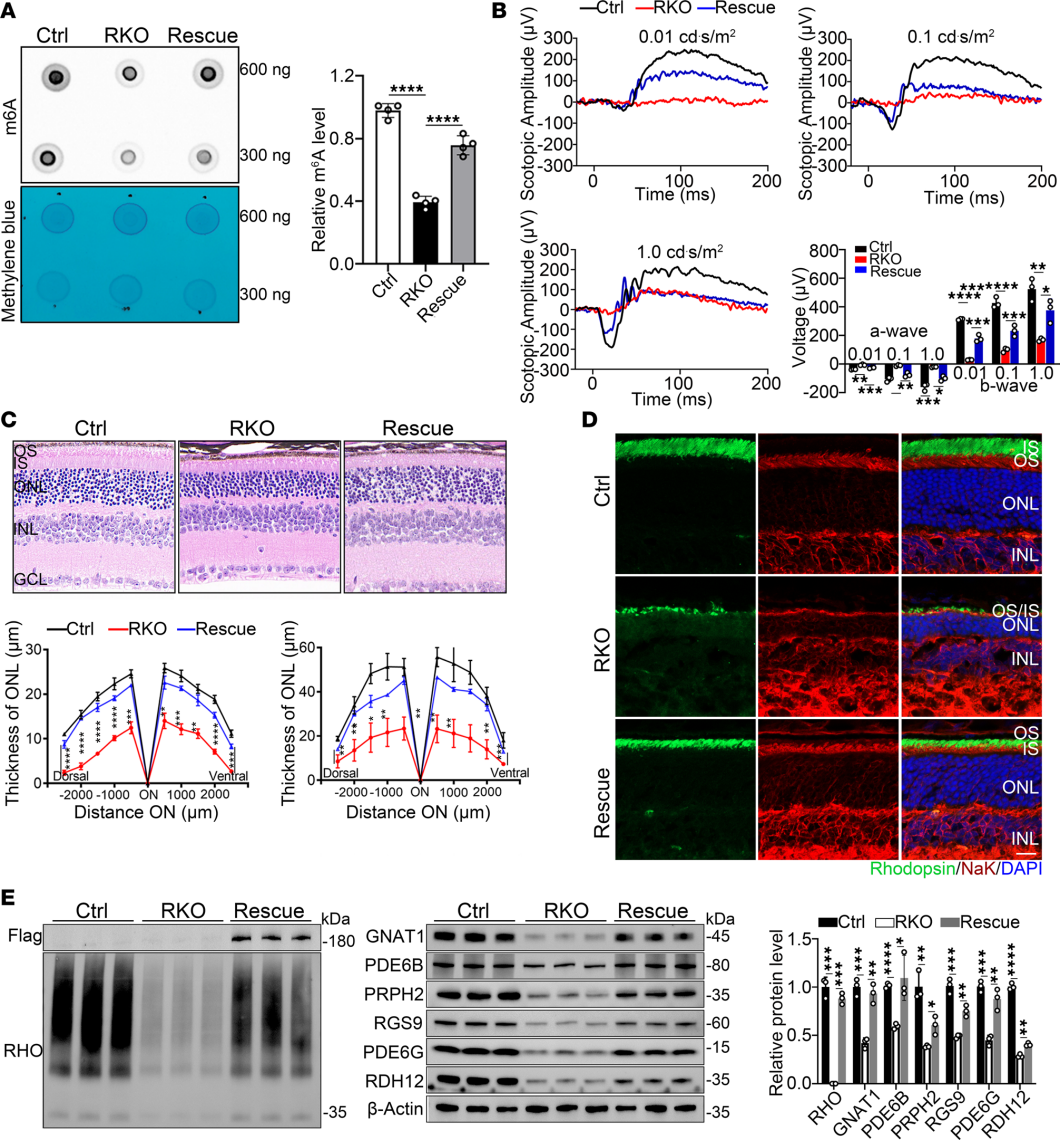
Replenishing *Virma* can attenuate photoreceptor degeneration in RKO mice. (**A**) m^6^A dot blot assay of global m^6^A abundance in retinas from 8-week-old Ctrl, RKO, and Rescue mice. Methylene blue staining was used as a loading control (1-way ANOVA, *n* = 4). (**B**) Representative scotopic ERG traces and statistical analysis at identified flash intensities in mice at 8 weeks of age (*n* = 3). Analyzed by 2-way ANOVA with post hoc Bonferroni’s multiple-comparisons test. (**C**) H&E staining of paraffin sections of RKO mice, Rescue mice, and Ctrl mice at 8 weeks, and quantification analysis of the photoreceptor segments (PRS) and ONL thickness of the groups (*n* = 3). Scale bar: 25 μm. Analyzed by multiple 2-tailed *t* tests with the Holm-Šídák method to correct for multiple comparisons. (**D**) Retina cryosections from 8-week-old mice were stained with OS marker rhodopsin and IS marker NaK ATPase. Scale bars: 25 μm. (**E**) Western blot and quantification analysis of visual perception–associated proteins (*n* = 3). OS, outer segment; IS, inner segment; ONL, outer nuclear layer; INL, inner nuclear layer; GCL, ganglion cell layer, RPS, photoreceptor segment. Data are presented as the mean ± SD. *P < 0.05; **P < 0.01; ***P < 0.001; ****P < 0.0001.

The m^6^A modification is a highly dynamic and reversible process, with both upregulation and downregulation of m^6^A modification reported to be involved in various cancers, suggesting that the m^6^A modification is crucial for the proper tissue function. We attempted to rescue the photoreceptor phenotypes in RKO mice by restoring m^6^A modification with a *Virma* overexpression allele (*Virma^loxp/loxp^;* CAG-*Virma-*3xFlag*;* *RHO-Cre*, hereafter named Rescue). Remarkably, reexpression of *Virma* in rods of RKO mice significantly improved visual perception and ameliorated degeneration of photoreceptors cells at 8 weeks of age (Figure 8). In *Virma*-rescued retinas, the global m^6^A methylation levels were significantly restored compared with those in RKO mice, as quantified by dot blot assay (Figure 8A). Scotopic ERG recordings revealed elevated amplitudes of a-waves and b-waves in Rescue mice compared with age-matched RKO mice, indicating the improved rod-driven circuit responses (Figure 8B). H&E staining confirmed the rescue effect on retinal morphology: Rescue mice showed significantly thickened photoreceptor segments and ONL compared with RKO mice, with values nearly restored to levels similar to those of Ctrl mice (Figure 8C). Immunofluorescence staining (Figure 8D) and Western blot analysis (Figure 8E) revealed increased expression of OS structural and functional proteins. In conclusion, these data provided additional evidence that VIRMA loss compromised photoreceptor function, highlighting the critical role of m^6^A methylation photoreceptor cells.

## Discussion

m^6^A methylation modification is the most abundant internal mRNA modification in mammals and plays a vital role in retina development and homeostasis maintenance. As a critical scaffold component, *VIRMA* guides region-selective m^6^A methylation by recruiting catalytic core components (32). Notably, the deletion of *VIRMA* led to the biggest reduction in m^6^A level but not in *METTL3/14* or *WTAP* knockdown cells (12, 36), highlighting its critical role in m^6^A modification. VIRMA has been reported to be involved in numerous malignant tumors (19, 40, 78–80) and nonneoplastic diseases, including cardiovascular system disease (81), respiratory system disease (82, 83), orthopedic diseases (84), reproductive system disease (41), and central nervous system diseases (37, 38). However, whether VIRMA, like other m^6^A writers (29, 30, 85–87), is involved in occurrence of neuron retina disease is yet to be elucidated. Given the link between m^6^A modification and retinal function, we generated and characterized rod- and cone-specific *Virma*-knockout mice (RKO and HKO) and showed that *Virma*-catalyzed m^6^A modification is essential for the function and survival of both rods and cones (Figures 1–3). When *Virma*-mediated m^6^A was depleted in rods, the whole profile m^6^A modification level of retina was dramatically decreased (Figure 4). Using an unbiased combined analysis of MeRIP-seq and RNA-seq data, we found that genes exhibiting simultaneous downregulation in both m^6^A modification and transcriptional levels in RKO mice were predominantly enriched in the visual perception cascade (Figures 4 and 5). Notably, RKO mice displayed visual impairment and photoreceptor loss following *Virma* depletion. Furthermore, reintroduction of *Virma* expression in rods successfully alleviated retinal lesions (Figure 8). Therefore, our results elucidate the key pathogenesis of *Virma*-associated retinal degeneration.

An interesting finding of our study is that the pathological phenotypes of retinal damage caused by *Virma* deficiency occur earlier and progress more rapidly than those resulting from deficiencies in other m^6^A writers, including core components *Mettl3* and *Mettl14* (Supplemental Figure 2). This finding highlights the crucial role of *VIRMA* in m^6^A methylation modification and is consistent with prior research (30). On one hand, this may be attributed to the downregulation of m^6^A modification levels on a larger number of target genes in the retina following *Virma* depletion. On the other hand, the nuclear speckle localization of *VIRMA* and its interaction with serine/arginine-rich splicing factor 3 (*SRSF3*) led us to investigate whether *VIRMA* regulates AS events in retina. SUPPA2 analysis demonstrated that *Virma* was involved in AS regulation in the retina, with the most pronounced changes in AS events after *Virma* deletion occurring primarily in A5SS, A3SS, SE, and RI. This analysis uncovered disparities in contrast to a previous study, which observed a higher percentage of SE events (78% in oocytes and 48% in HeLa cells) following *VIRMA* depletion (41). These discrepancies may be attributed to the unique role of *VIRMA* in different cell types and enhanced accuracy of data from high-throughput transcriptomic. After validation, we identified increased exon skipping in *Impdh1* and increased intron retention in *Pogl2* in the RKO mouse retinas (Figure 6). Previous studies have shown that IMPDH1 catalyzes the rate-limiting step of de novo guanine synthesis and involved in RP development. Furthermore, intron retention directly contributes to the regulation of gene expression and plays a particularly important role in the synaptic plasticity of neuronal cells (88). *Pogl2*, as the processivity subunit of the mtDNA polymerase **γ**, is solely responsible for mtDNA replication and has been associated with mtDNA depletion syndrome (89). Aberrant transcript appears and, consequently, mitochondrial dysfunction occurs in RKO mice, as illustrated in Figure 6. Furthermore, visual impairment and photoreceptor degeneration in rod-specific *Srsf3*-knockout mice provide additional evidence for the key role of stable splicing in retina homeostasis (Figure 7).

In summary, we demonstrated that *Virma* is required for photoreceptor function and survival, likely via the posttranslational regulation of visual perception signaling and AS in photoreceptors. With the decrease in m^6^A levels in the retina, *Virma* deficiency alters the expression pattern of visual perception cascade that orchestrates photoreceptor function, resulting in impaired visual function and photoreceptor degeneration. Reintroduction of *Virma* in rods can ameliorate *Virma*-associated retina degeneration. Notably, we found that *VIRMA* is localized in the nuclear speckles and interacts with *SRSF3*, implying an essential role of *VIRMA* in AS. Of importance, compared with those previously reported in oocytes and HeLa cells following *VIRMA* deletion, the differences in the proportion of significant AS event types in retina suggest the cell-specific role of *VIRMA* in mRNA metabolism. These data provide a comprehensive understanding of the role of *Virma* in retina and highlight the contribution of RNA epigenetic modification to nervous system function. Given the high conservation of *VIRMA* between mice and humans (>90% sequence homology, https://blast.ncbi.nlm.nih.gov/Blast.cgi), the findings from our model may be insightful for the development of future therapeutic strategies for related human retinal diseases. However, whether the homeostasis of m^6^A modification affects retinal function remains to be further explored.

## Methods

### Sex as a biological variable.

Sex was not considered as a biological variable. Both male and female mice were used in this study.

### Experimental animals.

Mice with *Virma* and *Srsf3* deletion specifically in retinal rod or cone cells were generated using the Cre-loxP system. The *Virma*-floxed mice and *Srsf3*-floxed mice in a C57BL/6J genomic background were constructed by Cyagen Biosciences. *RHO-Cre* mice were purchased from The Jackson Laboratory (stock no. 015850). The *HRGP-Cre* mice were a gift from Yunzheng Le at the University of Oklahoma Health Sciences Center, Norman, Oklahoma. *Virma*-floxed mice were mated with *RHO-Cre* or *HRGP-Cre* transgenic mice to obtain rod- or cone-specific *Virma-*knockout mice (named *Virma^flox/flox^*; *RHO*-Cre [RKO] or *Virma^flox/flox^*; *HRGP-Cre* [HKO] mice). To determine the specificity of Cre expression, we employed a tdTomato reporter allele (strain *Cg-Gt*(*ROSA*)26*Sortm14*(*CAG-tdTomato*)*Hze/J*; stock 007914). In this system, Cre-mediated recombination excises a loxp-flanked stop codon before the tdTomato expression cassette, allowing for the expression of tdTomato (red fluorescence) in Cre-positive cells.

Additionally, we acquired a knockin mouse model that carries the *SApolyA-CAG-LSL-Virma-3×Flag-IRES-EGFP-WPRE-pA* expression cassette inserted into the *Rosa26* locus on chromosome 6 (named *CAG-Virma*) from the Shanghai Model Organisms Center. *Virma* RKO mice were then mated with *CAG-Virma* mice to generate *Virma^flox/flox^*; *RHO*-Cre; CAG-*Virma* (named Rescue) mice. In the presence of Cre enzyme, the STOP cassette was removed, and Flag-tagged Virma was expressed for genetic rescue. Mouse genomic DNA samples were extracted from mouse tails and genotyped using the corresponding primers (Supplemental Table 2).

All animals were kept in specific pathogen–free grade rooms with controlled conditions: temperature at 25°C, relative humidity at approximately 40%–60%, and a 12-hour light/dark cycle.

### ERGs in mice.

ERG recordings were performed as described in a previous study (90). Briefly, mice were dark-adapted for at least 8 hours, and all subsequent procedures were performed under dim red light. Mice were anesthetized using ketamine (16 mg/kg body weight) and chlorpromazine (80 mg/kg body weight) mixed with saline. A drop of tropicamide was administered to the eyes of the mice to dilate the pupils. Dark-adapted ERGs in response to flashes of light with intensities ranging from 0.01 to 10.0 cd-s/m^2^ and light-adapted with intensities of 3.0 and 10.0 cd-s/m^2^ were recorded in mice using the Espion Visual Electrophysiology System (Diagnosys LLC).

### Histological analysis.

For H&E staining, eyes were extracted from mice of different ages and fixed overnight in FAS solution (0.08 M phosphate buffer containing 1.22% glutaraldehyde and 0.8% paraformaldehyde). Eyes were embedded in paraffin (in the same direction as the labeled embedding) and then cut to 5 μm sections. Sections containing the optic nerve were selected for H&E staining according to standard protocols.

### Immunohistochemistry.

For retinal section immunofluorescence, eyes were removed and fixed in 4 % PFA for 2 hours. After fixation, the cornea and lens were removed, and the eyes were dehydrated in 30% sucrose for 2 hours. The eyes were then embedded in OCT, and 10 μm thick sections were cut. Following permeabilization in PBS containing 5% FBS and 0.1% Triton X-100 for 1 hour, the sections were incubated overnight at 4°C with a primary antibody. After 3 washes in PBS, the sections were incubated for 2 hours at room temperature with Alexa Fluor 594/488–conjugated goat anti-mouse/rabbit secondary antibodies and DAPI. Images were acquired using a Zeiss LSM 900 confocal laser scanning microscope.

For retinal whole-mount staining, eyes were dissected and flattened as previously described (91). After fixing, permeabilization, and staining, the whole mounts were visualized with a Zeiss LSM 900 confocal laser scanning microscope. The primary antibodies used for immunohistochemistry are shown in Supplemental Table 3.

### Western blotting.

Retinas or cells were lysed in RIPA buffer (89900, Thermo) with a mixture of protease and phosphatase inhibitors. After sonication and centrifugation, the supernatant was diluted with 4x SDS loading buffer. Equal amounts of protein were separated on SDS-PAGE gels and transferred to NC membranes. The membranes were incubated using 8% skim milk in Tris buffer containing 0.1% Tween 20 (TBST) at room temperature for 1 hour and then primary antibodies were added overnight at 4°C. The membranes were washed 3 times in TBST and then incubated with horseradish peroxidase–coupled antibody at room temperature for 2 hours. Signals were developed using SuperSignal West Pico Chemiluminescent Substrate (Thermo Fischer). ImageJ (NIH) was employed to determine the relative protein density. Relative quantification was performed using β-actin as an internal reference. The primary antibodies used for Western blotting are shown in Supplemental Table 2.

### Light-dark box test.

The device used for the light-dark box test (XR-XB110, XINRUN) consisted of a box divided into a dark compartment and an illuminated compartment with a luminance of 2,000 lux, connected to the acquisition device through a small opening. Mice were first placed in the light compartment with its back to the opening and moved freely between the 2 compartments for 5 minutes. Based on the behavior recorded by the system’s video camera, the time spent in the light and dark compartments, the distance traveled, the total number of transitions, and the average speed of the mice were automatically analyzed using VisuTrack software. After each experiment, the compartments were cleaned with 75% ethanol to remove olfactory cues.

### Optomotor response test.

The optomotor response test was performed as described in previous study (53). Briefly, mice were placed on an elevated platform in the center to move freely and were presented with vertical sinusoidal grating stimuli, rotated horizontally in either a clockwise or counterclockwise direction, which was randomly generated by virtual cylinders projected onto 4 surrounding LCD displays. The visual stimuli were driven at a spatial frequency of 0.2 cycles/°. Head movements triggered by the visual stimuli were recorded by a video camera placed above the animal and analyzed by an algorithm that automatically tracked the mouse head position (OptoTrack XR-OT101 system, OptoTrack Version 4, XINRUN). Each mouse was tested at least 10 times, and the average of its performance was taken for analysis. After each mouse was tested, the compartment was cleaned with 75% ethanol to remove olfactory cues.

### m^6^A dot blot assay.

Total RNA was extracted from mouse retinas, and the mRNA was purified using PolyATtract mRNA Isolation System III (Z5300, Promega) following the standard procedure. Following adjustment of mRNA concentrations to uniform levels, both experimental and control groups were denatured at 95°C for 2 minutes. Subsequently, 2 μL mRNA was transferred to a nitrocellulose (NC) membrane, which was air-dried at room temperature and crosslinked under 1,200 W UV light for 50 seconds. The membrane was then washed with TBST buffer for 5 minutes, followed by 0.02% methylene blue staining to ensure consistency across the groups, and the resulting images were recorded. To block nonspecific binding, the membrane was incubated with 5% BSA for 1 hour at room temperature. It was then probed with an m^6^A antibody overnight at 4°C. The following day, the membrane was washed 3 times with TBST and incubated with a horseradish peroxidase–conjugated secondary antibody for 2 hours. The images were captured using an e-BLOT imager.

### RNA-seq and m^6^A MeRIP-seq.

For RNA-seq, retinas from 1-month-old Ctrl and RKO mice were harvested, and total RNA was extracted. mRNA was then purified and fragmented into 60–200 nt segments, followed by library construction. Sequencing was performed on the Illumina NovaSeq 6000 platform, and differential expression analysis was conducted using the DESeq2 R package. Gene expression values were transformed to log_2_ scale for further analysis, with *P* ≤ 0.05 considered statistically significant.

For MeRIP-seq, retinas from 1-month-old Ctrl and RKO mice were also collected, and total RNA was extracted. The RNA quality and integrity were assessed, and the RNA was fragmented into 200 nt segments. The fragmented RNA was then incubated with an m^6^A antibody for 2 hours at 4°C to perform immunoprecipitation. Library construction was carried out, and sequencing was performed on the Illumina NovaSeq 6000 platform using 3 independent biological replicates. The reads per kilobase per million mapped reads values for the 5′ UTR, coding sequence (CDS), and 3′ UTR of all genes were calculated using deepTools software. Differential peaks were identified using the ExomePeak R package, with *P* ≤ 0.05 considered statistically significant.

### MeRIP-qPCR and RT-qPCR.

Total RNA was extracted from 1-month-old Ctrl and RKO mice using TRIzol (ET111-01, TransGen). The MeRIP assay was carried out with a GenSeq m^6^A MeRIP kit (GS-ET-001, CloudSeq) in accordance with the manufacturer’s instruction. Genes were quantified for m^6^A modification level by RT-qPCR using specific primers, and gene expression level also quantified by RT-qPCR using specific primers (Supplemental Table 4).

### Gene knockdown and overexpression strategies.

HEK-293T cells (purchased from ATCC) were seeded onto 6 cm dishes and cultured overnight to allow cell attachment. The following day, the cells were transfected with lentivirus containing human *VIRMA* shRNA (5′-AAGACCTTCGTGAAGTATA-3′, Genechem) or a negative control shRNA (5′-TTCTCCGAACGTGTCACGT-3′). After 72 hours of culture, puromycin selection was applied to isolate successfully transfected cells, which were subsequently used for further experiments.

For exogenous protein overexpression, HEK-293T cells were seeded onto 6 cm dishes and cultured overnight for attachment. The next day, cells were transfected with corresponding plasmids using Lipofectamine 3000 (L3000015, Invitrogen) following the manufacturer’s protocol, including pTriEx 1.1-Neo-*VIRMA*-3×Flag and pcDNA3.1-*SRSF3*-HA plasmids. The pTriEx 1.1-Neo-*VIRMA*-3×Flag plasmid was a gift from Jianzhao Liu (Zhejiang University, Zhejiang, China) and Chuan He (Howard Hughes Medical Institute, The University of Chicago, Chicago, Illinois, USA). After 36–48 hours of incubation, the cells were harvested for subsequent experiments.

### Statistics.

GraphPad Prism 8.0 software was used for statistical analysis. All data are presented as mean ± SD. The data sets were tested for normal distribution using Shapiro-Wilk test. Statistical significance was determined by unpaired 2-tailed Student’s *t* test or by 1- or 2-way ANOVA followed by Tukey’s, Dunnett’s, or Šídák’s multiple-comparisons test as appropriate. At least 3 independent experiments were performed. Results were considered significant when *P* < 0.05.

### Study approval.

All experimental protocols were approved by the Ethics Committee of Sichuan Provincial People’s Hospital (LS-2023-046) and adhered to the ARVO Statement for the Use of Animals in Ophthalmic and Vision Research.

### Data availability.

All data produced or analyzed in the present study are included in the manuscript and supplemental files. MeRIP-seq and high-throughput transcriptomic data have been uploaded to the Genome Sequence Archive (https://ngdc.cncb.ac.cn/gsa/) under accession numbers CRA036966 and CRA036993, respectively. The proteomics data have been deposited to the ProteomeXchange Consortium (https://proteomecentral.proteomexchange.org) with the dataset identifier PXD073210. Values for all data points in graphs are reported in the Supporting Data Values file. All data needed to evaluate the conclusions in the manuscript are present in the manuscript and/or the supplemental materials.

## Author contributions

Conception and design were contributed by XZ, LZ, JH, and LY. Acquisition of data was contributed by WL, XW, RZ, FZ, YF, and KS. Analysis, interpretation of data, and generation of figures were contributed by WL, XW, RZ, FZ, LZ, and XZ. Funding acquisition and writing, review, and revision of the manuscript were contributed by WL, KS, LZ, and XZ. Study supervision was contributed by XZ. All authors reviewed and approved the manuscript.

## Conflict of interest

FZ is a current employee of Beijing Chinagene Co. LTD.

## Funding support

- National Natural Science Foundation of China (82371083 to XZ, 82121003 to XZ, 82501317 to LW, 82501312 to KS).
- Department of Science and Technology of Sichuan Province (2023ZYD0172 to XZ).
- Research grant from Jinfeng Laboratory (JFLKYXM202403AZ-101 to XZ).

## Supplementary Material

Supplemental data

Unedited blot and gel images

Supplemental table 1

Supporting data values

## Version 1 03/20/2026

In-Press Preview

## Version 2 05/08/2026

Electronic publication

## References

[1] Ebrey T (2001). Vertebrate photoreceptors. Prog Retin Eye Res.

[2] Liu X (2021). Molecular diagnosis based on comprehensive genetic testing in 800 Chinese families with non-syndromic inherited retinal dystrophies. Clin Exp Ophthalmol.

[3] Schneider N (2022). Inherited retinal diseases: Linking genes, disease-causing variants, and relevant therapeutic modalities. Prog Retin Eye Res.

[4] Olivares-González L (2021). Retinal inflammation, cell death and inherited retinal dystrophies. Int J Mol Sci.

[5] Campochiaro PA (2018). The mechanism of cone cell death in Retinitis Pigmentosa. Prog Retin Eye Res.

[6] Pagon RA (1988). Retinitis pigmentosa. Surv Ophthalmol.

[7] Hartong DT (2006). Retinitis pigmentosa. Lancet.

[8] Cavalli G (2019). Advances in epigenetics link genetics to the environment and disease. Nature.

[9] Zhang L (2020). Epigenetics in health and disease. Adv Exp Med Biol.

[10] Dominissini D (2012). Topology of the human and mouse m6A RNA methylomes revealed by m6A-seq. Nature.

[11] Zhao BS (2017). Post-transcriptional gene regulation by mRNA modifications. Nat Rev Mol Cell Biol.

[12] Liu J (2014). A METTL3-METTL14 complex mediates mammalian nuclear RNA N6-adenosine methylation. Nat Chem Biol.

[13] Pendleton KE (2017). The U6 snRNA m^6^A methyltransferase METTL16 regulates SAM synthetase intron retention. Cell.

[14] Patil DP (2016). m(6)A RNA methylation promotes XIST-mediated transcriptional repression. Nature.

[15] Wen J (2018). Zc3h13 regulates nuclear RNA m^6^A methylation and mouse embryonic stem cell self-renewal. Mol Cell.

[16] Jia G (2011). N6-methyladenosine in nuclear RNA is a major substrate of the obesity-associated FTO. Nat Chem Biol.

[17] Zheng G (2013). ALKBH5 is a mammalian RNA demethylase that impacts RNA metabolism and mouse fertility. Mol Cell.

[18] Bawankar P (2021). Hakai is required for stabilization of core components of the m^6^A mRNA methylation machinery. Nat Commun.

[19] Lan T (2019). KIAA1429 contributes to liver cancer progression through N6-methyladenosine-dependent post-transcriptional modification of GATA3. Mol Cancer.

[20] Mendel M (2018). Methylation of structured RNA by the m^6^A writer METTL16 is essential for mouse embryonic development. Mol Cell.

[21] Shi H (2019). Where, when, and how: context-dependent functions of RNA methylation writers, readers, and erasers. Mol Cell.

[22] Alarcón CR (2015). HNRNPA2B1 is a mediator of m(6)A-dependent nuclear RNA processing events. Cell.

[23] Huang H (2018). Recognition of RNA N^6^-methyladenosine by IGF2BP proteins enhances mRNA stability and translation. Nat Cell Biol.

[24] Shi Y (2019). YTHDF1 links hypoxia adaptation and non-small cell lung cancer progression. Nat Commun.

[25] Bartosovic M (2017). N6-methyladenosine demethylase FTO targets pre-mRNAs and regulates alternative splicing and 3’-end processing. Nucleic Acids Res.

[26] Chen L (2022). Nuclear m^6^ A reader YTHDC1 suppresses proximal alternative polyadenylation sites by interfering with the 3’ processing machinery. EMBO Rep.

[27] Frye M (2016). Post-transcriptional modifications in development and stem cells. Development.

[28] Liu J (2019). Regulation of gene expression by N^6^-methyladenosine in cancer. Trends Cell Biol.

[29] Xin Y (2022). m^6^A epitranscriptomic modification regulates neural progenitor-to-glial cell transition in the retina. Elife.

[30] Yang Y (2022). Mettl14-mediated m6A modification is essential for visual function and retinal photoreceptor survival. BMC Biol.

[31] Wang Y (2024). m6A-mediated upregulation of imprinted in Prader-Willi syndrome induces aberrant apical-basal polarization and oxidative damage in RPE cells. Invest Ophthalmol Vis Sci.

[32] Yue Y (2018). VIRMA mediates preferential m6A mRNA methylation in 3′UTR and near stop codon and associates with alternative polyadenylation. Cell Discov.

[33] Zhang C (2022). Gene amplification-driven RNA methyltransferase KIAA1429 promotes tumorigenesis by regulating BTG2 via m6A-YTHDF2-dependent in lung adenocarcinoma. Cancer Commun (Lond).

[34] Ping XL (2014). Mammalian WTAP is a regulatory subunit of the RNA N6-methyladenosine methyltransferase. Cell Res.

[35] Schwartz S (2014). Perturbation of m6A writers reveals two distinct classes of mRNA methylation at internal and 5’ sites. Cell Rep.

[36] Han D (2022). Dynamic assembly of the mRNA m6A methyltransferase complex is regulated by METTL3 phase separation. PLoS Biol.

[37] Zou D (2024). Single-cell and spatial transcriptomics reveals that PTPRG activates the m^6^A methyltransferase VIRMA to block mitophagy-mediated neuronal death in Alzheimer’s disease. Pharmacol Res.

[38] Hong H (2024). VIRMA promotes neuron apoptosis via inducing m6A methylation of STK10 in spinal cord injury animal models. CNS Neurosci Ther.

[39] Liu ZC (2022). KIAA1429 regulates alternative splicing events of cancer-related genes in hepatocellular carcinoma. Front Oncol.

[40] Qian JY (2019). KIAA1429 acts as an oncogenic factor in breast cancer by regulating CDK1 in an N6-methyladenosine-independent manner. Oncogene.

[41] Hu Y (2020). Oocyte competence is maintained by m^6^A methyltransferase KIAA1429-mediated RNA metabolism during mouse follicular development. Cell Death Differ.

[42] Modrek B (2001). Genome-wide detection of alternative splicing in expressed sequences of human genes. Nucleic Acids Res.

[43] Yeo G (2004). Variation in alternative splicing across human tissues. Genome Biol.

[44] Cao H (2011). Temporal and tissue specific regulation of RP-associated splicing factor genes PRPF3, PRPF31 and PRPC8--implications in the pathogenesis of RP. PLoS One.

[45] Aísa-Marín I (2021). The alter retina: alternative splicing of retinal genes in health and disease. Int J Mol Sci.

[46] Keuthan CJ (2023). Alternative RNA splicing in the retina: insights and perspectives. Cold Spring Harb Perspect Med.

[47] Liu MM (2013). Alternative splicing and retinal degeneration. Clin Genet.

[48] Yang Y (2024). The m^6^A reader YTHDC2 maintains visual function and retinal photoreceptor survival through modulating translation of PPEF2 and PDE6B. J Genet Genomics.

[49] Jiang X (2024). Mettl3-mediated m6A modification is essential for visual function and retinal photoreceptor survival. Invest Ophthalmol Vis Sci.

[50] Li S (2005). Rhodopsin-iCre transgenic mouse line for Cre-mediated rod-specific gene targeting. Genesis.

[51] Le YZ (2004). Targeted expression of Cre recombinase to cone photoreceptors in transgenic mice. Mol Vis.

[52] Macosko EZ (2015). Highly parallel genome-wide expression profiling of individual cells using nanoliter droplets. Cell.

[53] Qin H (2023). Vision rescue via unconstrained in vivo prime editing in degenerating neural retinas. J Exp Med.

[54] Arroba AI (2018). IGF-1, inflammation and retinal degeneration: a close network. Front Aging Neurosci.

[55] Vidal L (2010). Reaction of Müller cells in an experimental rat model of increased intraocular pressure following timolol, latanoprost and brimonidine. Brain Res Bull.

[56] Bringmann A (2001). Role of Muller cells in retinal degenerations. Front Biosci.

[57] Sahel J (2010). Retinitis pigmentosa and other dystrophies. Dev Ophthalmol.

[58] Lamb TD (2022). Photoreceptor physiology and evolution: cellular and molecular basis of rod and cone phototransduction. J Physiol.

[59] Fu Y (2014). Gene expression regulation mediated through reversible m^6^A RNA methylation. Nat Rev Genet.

[60] Meyer KD (2012). Comprehensive analysis of mRNA methylation reveals enrichment in 3′ UTRs and near stop codons. Cell.

[61] Tripathi V (2012). SRSF1 regulates the assembly of pre-mRNA processing factors in nuclear speckles. Mol Biol Cell.

[62] More DA (2020). SRSF3: Newly discovered functions and roles in human health and diseases. Eur J Cell Biol.

[63] Xiao W (2016). Nuclear m(6)A reader YTHDC1 regulates mRNA splicing. Mol Cell.

[64] Trincado JL (2018). SUPPA2: fast, accurate, and uncertainty-aware differential splicing analysis across multiple conditions. Genome Biol.

[65] Bowne SJ (2006). Why do mutations in the ubiquitously expressed housekeeping gene IMPDH1 cause retina-specific photoreceptor degeneration?. Invest Ophthalmol Vis Sci.

[66] Kennan A (2002). Identification of an IMPDH1 mutation in autosomal dominant retinitis pigmentosa (RP10) revealed following comparative microarray analysis of transcripts derived from retinas of wild-type and Rho(–/–) mice. Hum Mol Genet.

[67] Grover S (2004). A novel IMPDH1 mutation (Arg231Pro) in a family with a severe form of autosomal dominant retinitis pigmentosa. Ophthalmology.

[68] Yap K (2012). Coordinated regulation of neuronal mRNA steady-state levels through developmentally controlled intron retention. Genes Dev.

[69] Yao J (2020). Prevalent intron retention fine-tunes gene expression and contributes to cellular senescence. Aging Cell.

[70] Ferraris S (2008). Progressive external ophthalmoplegia and vision and hearing loss in a patient with mutations in POLG2 and OPA1. Arch Neurol.

[71] Zong Y (2024). Mitochondrial dysfunction: mechanisms and advances in therapy. Signal Transduct Target Ther.

[72] Gorman GS (2016). Mitochondrial diseases. Nat Rev Dis Primers.

[73] Lopriore P (2022). Mitochondrial epilepsy, a challenge for neurologists. Int J Mol Sci.

[74] Chakarova CF (2002). Mutations in HPRP3, a third member of pre-mRNA splicing factor genes, implicated in autosomal dominant retinitis pigmentosa. Hum Mol Genet.

[75] Vithana EN (2001). A human homolog of yeast pre-mRNA splicing gene, PRP31, underlies autosomal dominant retinitis pigmentosa on chromosome 19q13.4 (RP11). Mol Cell.

[76] McKie AB (2001). Mutations in the pre-mRNA splicing factor gene PRPC8 in autosomal dominant retinitis pigmentosa (RP13). Hum Mol Genet.

[77] Wan J (2011). Dynamic usage of alternative splicing exons during mouse retina development. Nucleic Acids Res.

[78] Miao R (2020). KIAA1429 regulates cell proliferation by targeting c-Jun messenger RNA directly in gastric cancer. J Cell Physiol.

[79] Li N (2023). KIAA1429/VIRMA promotes breast cancer progression by m^6^ A-dependent cytosolic HAS2 stabilization. EMBO Rep.

[80] Xu Y (2021). VIRMA contributes to non-small cell lung cancer progression via N6-methyladenosine-dependent DAPK3 post-transcriptional modification. Cancer Lett.

[81] Wang P (2021). KIAA1429 and ALKBH5 oppositely influence aortic dissection progression via regulating the maturation of Pri-miR-143-3p in an m6A-dependent manner. Front Cell Dev Biol.

[82] Fei L (2022). The effect of N6-methyladenosine (m6A) factors on the development of acute respiratory distress syndrome in the mouse model. Bioengineered.

[83] Dai B (2021). Significance of RNA N6-methyladenosine regulators in the diagnosis and subtype classification of childhood asthma using the gene expression omnibus database. Front Genet.

[84] Zhu B (2021). Comprehensive analysis of N6-methyladenosine (m^6^A) modification during the degeneration of lumbar intervertebral disc in mice. J Orthop Translat.

[85] Li L (2023). Mettl14-mediated m^6^A modification ensures the cell-cycle progression of late-born retinal progenitor cells. Cell Rep.

[86] Suo L (2022). METTL3-mediated *N*^6^-methyladenosine modification governs pericyte dysfunction during diabetes-induced retinal vascular complication. Theranostics.

[87] Anderson AM (2014). The Drosophila Wilms’ Tumor 1-Associating Protein (WTAP) homolog is required for eye development. Dev Biol.

[88] Grabski DF (2021). Intron retention and its impact on gene expression and protein diversity: A review and a practical guide. Wiley Interdiscip Rev RNA.

[89] Rahman S (2019). POLG-related disorders and their neurological manifestations. Nat Rev Neurol.

[90] Li X (2023). Deletion of Emc1 in photoreceptor cells causes retinal degeneration in mice. FEBS J.

[91] Ma S (2015). Loss of mTOR signaling affects cone function, cone structure and expression of cone specific proteins without affecting cone survival. Exp Eye Res.

