# Supplementary material for: VIRMA modulates function of photoreceptor cells through m6A modification and alternative splicing
Source: JCI Insight. 2026 Mar 19;11(9):e197880. doi: 10.1172/jci.insight.197880 (PMC13232022; doi:10.1172/jci.insight.197880)
Supplement: Supplemental data [file jciinsight-11-197880-s187.pdf]

VIRMA modulates function of photoreceptor cells through m^{6}A modification and alternative splicing

Wenjing Liu[1,2#], Xiaojing Wu[1#], Rong Zou[2#], Fan Zhang[3#], Yudi Fan[2], Kuanxiang Sun[2], Liping Yang[4*], Jiang Hu[1*], Lin Zhang[2,5*], Xianjun Zhu[1,2,5*]

Supplementary data include Supplemental Figure 1-9 and Supplementary Table 2-4.

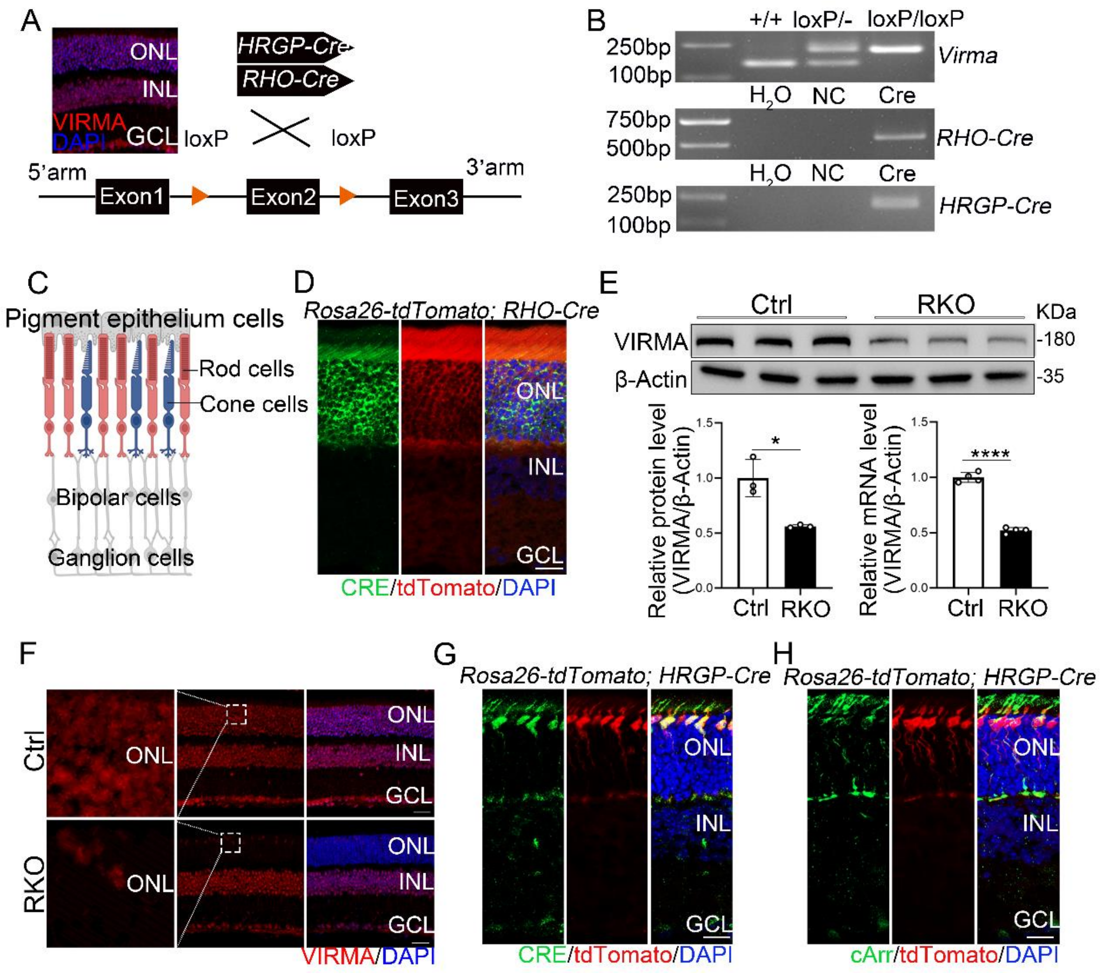

Supplemental Figure 1. Generation of the photoreceptor specific *Virma* knockout mice. (**A**) Representative images of VIRMA (red) expression in the retina and schematic drawings of the genetic deletion strategy for *Virma* in rods and cones using

RHO-Cre and *HRGP-Cre*, respectively. Scale bars, 25 μm. (**B**) Genotyping of RKO and HKO mice. (**C**) Schematic diagram of mice retina. (**D**) Immunofluorescence staining of retinal section from *Rosa26-td*Tomato; *RHO-Cre* mice with anti-Cre antibody (green). Scale bar, 50 μm. (**E**) Western blot and RT-qPCR comparison of VIRMA expression in retinas of Ctrl and RKO mice (Student's *t* test, n=3). (**F**) Immunofluorescence staining of retinal sections with VIRMA antibody (red) from Ctrl and RKO mice at 3 weeks of age. Scale bars, 25 μm. (**G**) Immunofluorescence staining of retinal section from *Rosa26-td*Tomator; *HRGP-Cre* mice with anti-Cre antibody (green). Scale bar, 50 μm. (**H**) The retina section was labeled with cArr antibody (green) to mark cone photoreceptor cells. Scale bar, 50 μm. ONL, outer nuclear layer; INL, inner nuclear layer; GCL, ganglion cell layer. Data are presented as the mean ± SD. *p < 0.05; ****p < 0.0001; #, no significant difference.

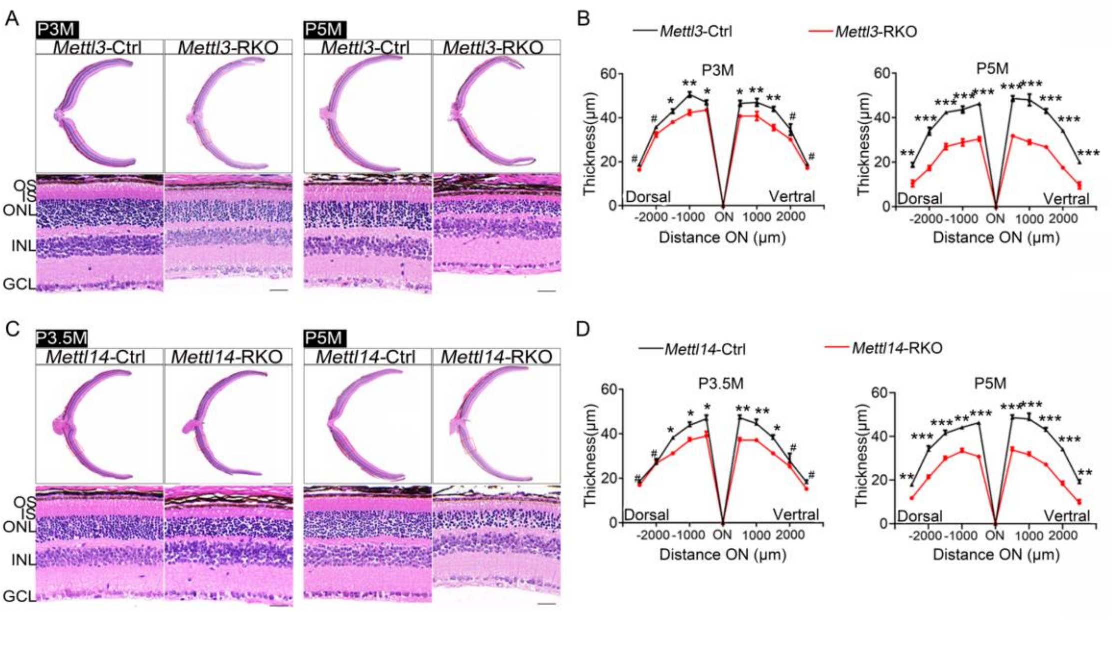

Supplemental Figure 2. Photoreceptor degeneration in *Mettl3/14* rods-specific

knockout mice. (**A**) H&E staining of paraffin sections of *Mettl3*-Ctrl and *Mettl3*-RKO retinas at the ages of 3 months and 5 months, respectively. Scale bars, 50 μm. (**B**) Quantitative analysis of the ONL thickness in the *Mettl3*-Ctrl and *Mettl3*-RKO retinas at defined ages (n = 3). (**C**) H&E staining of paraffin sections of *Mettl14*-Ctrl and *Mettl14*-RKO retinas at the ages of 3.5 months and 5 months, respectively. Scale bars, 50 μm. (**D**) Quantitative analysis of the ONL thickness in the *Mettl14*-Ctrl and *Mettl14*-RKO retinas at defined ages (n = 3). OS, outer segment; IS, inner segment; ONL, outer nuclear layer; INL, inner nuclear layer; GCL, ganglion cell layer. Data are presented as the mean ± SD. ONL thickness was analyzed by multiple 2-tailed *t* tests with the Holm-Sidak method to correct for multiple comparisons. *p < 0.05; **p < 0.01; ***p < 0.001; ****p < 0.0001; #, no significant difference.

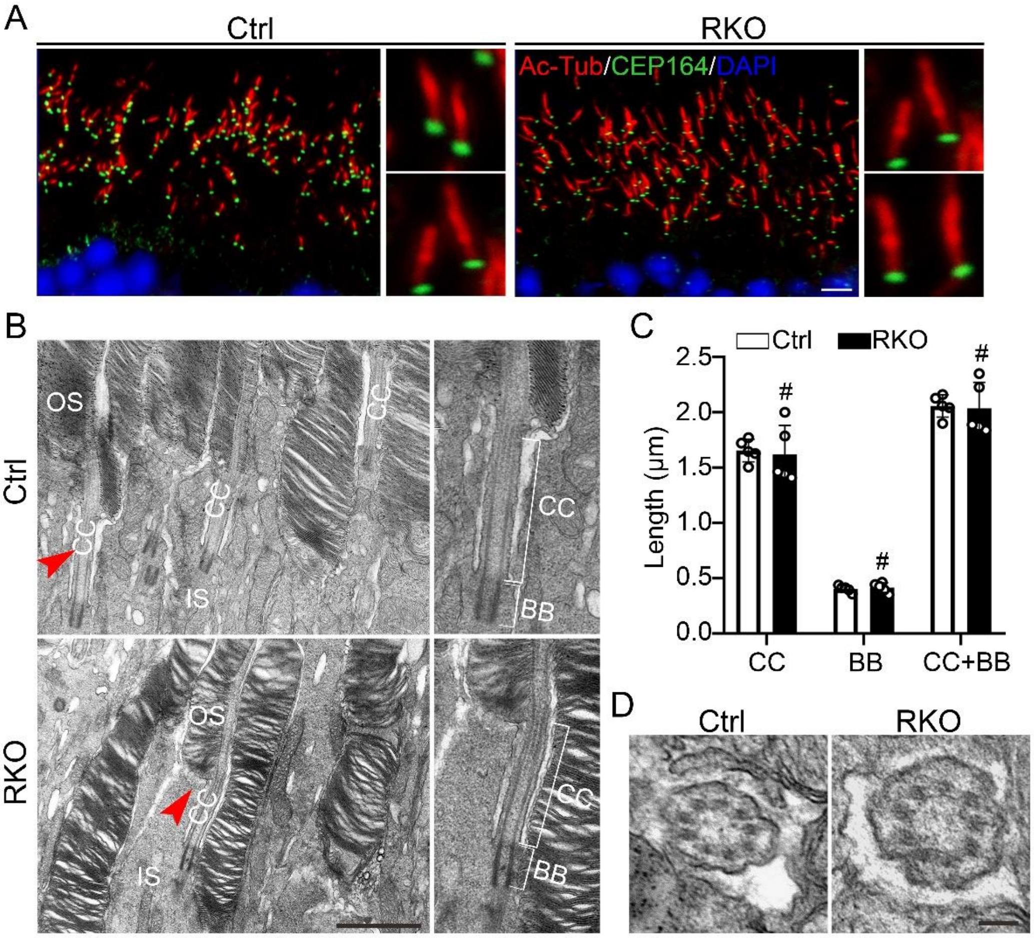

Supplemental Figure 3. No visible defect was observed in cilia structure of RKO mice. (**A**) Representative immunofluorescence images with a ciliary marker acetylated α-tubulin (red) and the centriole marker CEP164 (green) in retinas from 4-week-old Ctrl and RKO mice. Scale bars, 2 μm and 1 μm (higher-magnification images). (**B**) Representative electron micrographs of the ciliary region of photoreceptor cells from 4-week-old Ctrl and RKO mice. Scale bars, 2 μm and 1 μm (higher-magnification images). (**C**) Quantitative analyses of the length of the BB and CC in Ctrl and RKO photoreceptors (Student's *t* test, n= 5). OS, outer segment; IS, inner segment; CC, connecting cilium; BB, basal body. (**D**) Representative electron micrographs of the transverse section of cilia. Scale bars, 200 nm. Data are presented as the mean ± SD. #,

no significant difference.

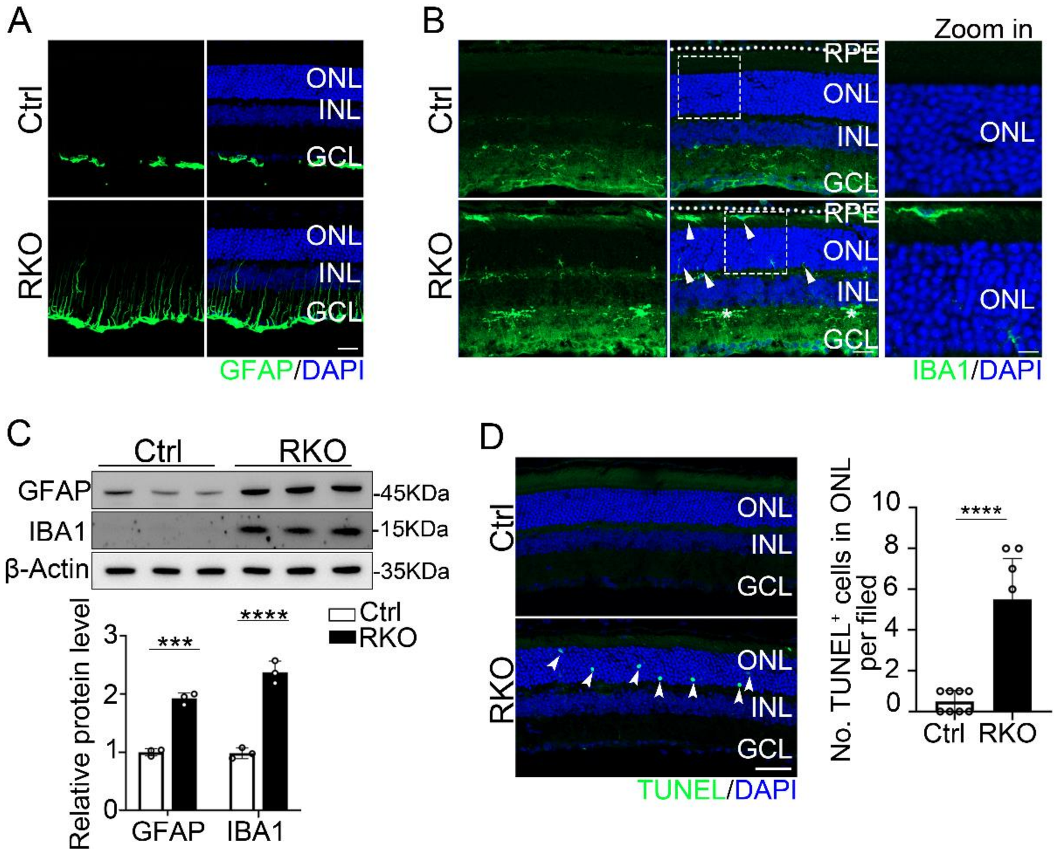

Supplemental Figure 4. Activation of resident retinal glial and immune cells in RKO mice. (**A**) Retina cryosections from 4-week-old mice were stained with GFAP (green). Scale bars, 20 µm. (**B**) Retina cryosections from 4-week-old mice were labeled with IBA1 (green). White arrowheads indicate IBA1-positive cells located inside or at the RPE (basal) side of the RKO retina. White dashed lines indicate RPE. Scale bars, 20 µm and 5µm (higher-magnification images). (**C**) Western blot and quantitative analysis of retina lysates from 4-week-old Ctrl and RKO mice (Student's *t* test, n=3). (**D**) Immunofluorescence labelling of retina cryosections from 4-week-old mice with the TUNEL assay kit and quantification of the number of TUNEL-positive cell (green) in ONL per field (Student's *t* test, n=8). White arrowheads represent TUNEL-positive

cells. Scale bars, 20 μm. Data are presented as the mean ± SD. ***$p < 0.001$; ****$p < 0.0001$.

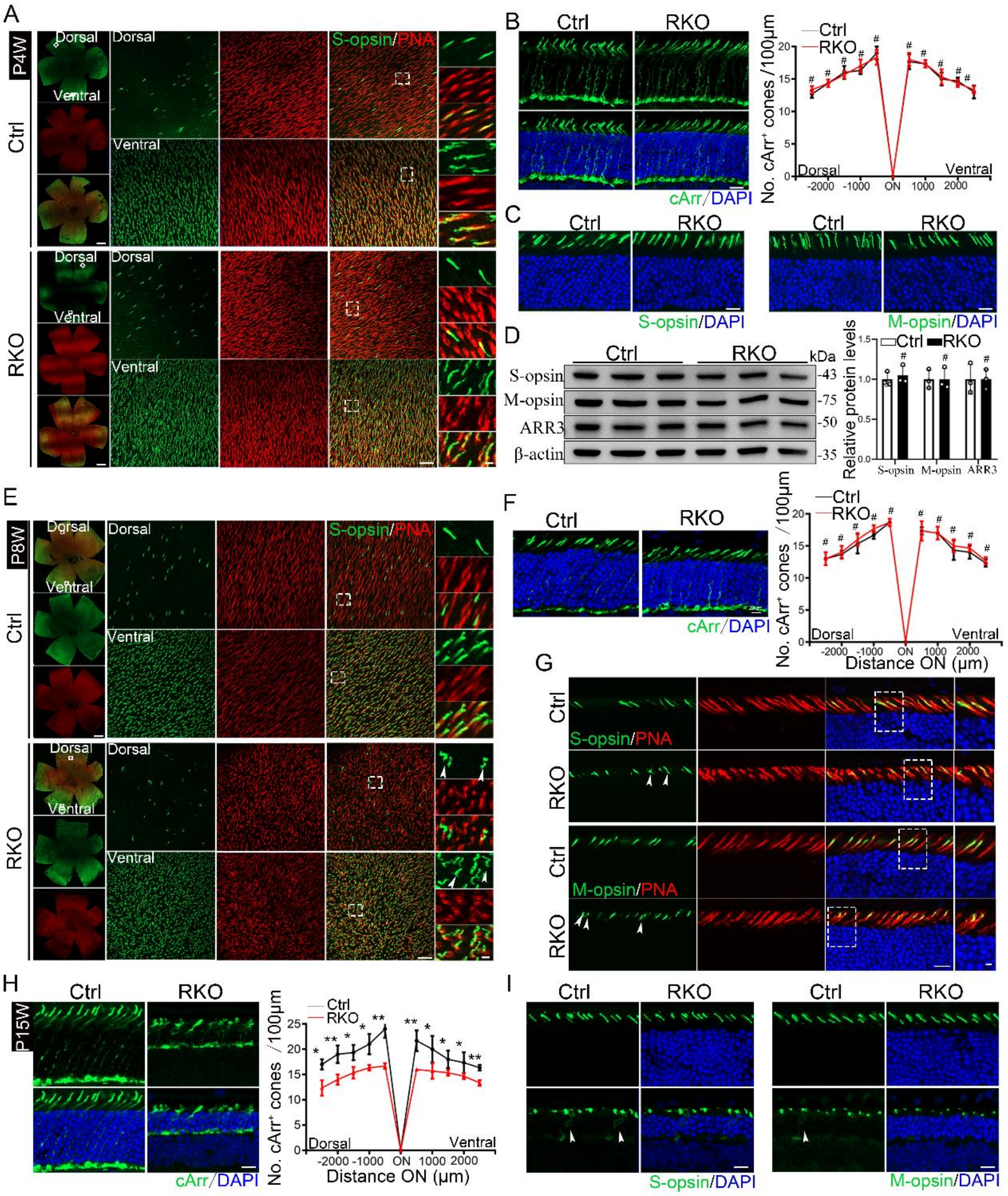

Supplemental Figure 5. Cone degeneration followed rods loss in RKO mice. (A) Immunostaining of retina flat-mount from 4-week-old Ctrl and RKO mice with S-opsin (greed) and PNA (red). Scale bars, 50 μm. Representative images from the dorsal and

ventral retinal quadrant are shown. Scale bars, 20 μm. Inset images showed a cropped and zoomed image. Scale bars, 5 μm. (**B**) Representative immunofluorescence images of cArr (green) of retina sections from 4-week-old Ctrl and RKO mice and the number of cArr-positive cones per 500 μm field was quantified. Scale bars, 25 μm. (**C**) Retinal cryosections from 4-week-old Ctrl and RKO mice were labeled with S-opsin and M-opsin. Scale bars, 20 μm. (**D**) Western blot and quantification analysis of cone related proteins in 4-week-old Ctrl and RKO mice (Student's *t* test, n=3). (**E**) Immunostaining of retina flat-mount from 8-week-old Ctrl and RKO mice with S-opsin (greed) and PNA (red), and representative images were presented. White arrowheads indicate the misshaped cones. Scale bars, 50 μm (retina flat-mount), 20 μm (dorsal and ventral retinal quadrant image), and 5 μm (zoomed image). (**F**) Representative immunofluorescence images and statical analysis of cone numbers from 8-week-old Ctrl and RKO mice. Scale bars, 25 μm. (**G**) Retinal cryosections from 8-week-old Ctrl and RKO mice were labeled with S/M-opsin and PNA. White arrowheads indicate the misshaped cones. Scale bars, 20 μm and 1 μm (zoomed images). (**H**) Representative immunofluorescence images and statical analysis of cone numbers from 15-week-old Ctrl and RKO mice. Scale bars, 25 μm. (**I**) Retinal cryosections from 15-week-old Ctrl and RKO mice were labeled with S-opsin and M-opsin. White arrowheads indicate mislocalized S/M-opsin. Scale bars, 20 μm. Data are presented as the mean ± SD. The number of cArr-positive cone cells was analyzed by multiple 2-tailed *t* tests with the Holm-Sidak method to correct for multiple comparisons. $^{*}p < 0.05$; $^{**}p < 0.01$; #, no significant difference.

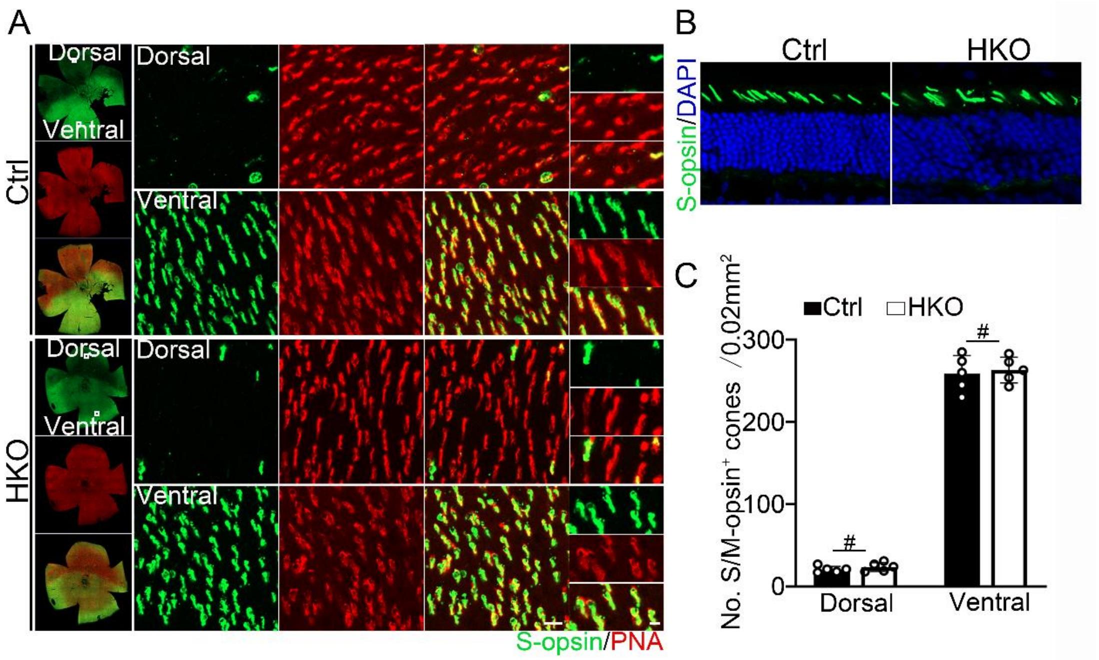

Supplemental Figure 6. Distortion of S-opsin-positive cones in HKO mice. (**A**) Immunostaining of retina flat-mount from 8-week-old Ctrl and HKO mice with S-opsin (greed) and PNA (red). Scale bars, 50 µm. Representative images from the dorsal and ventral retinal quadrant are shown. Scale bars, 20 µm. Inset images showed a cropped and zoomed image. Scale bars, 5 µm. (**B**) Retinal cryosections from 8-week-old Ctrl and HKO mice were labeled with S-opsin. Scale bars, 20 µm. (**C**) Quantification of the number of S-opsin-positive cones at both dorsal and ventral side of the retain per field (Student's *t* test, n=5). Data are presented as the mean ± SD. #, no significant difference.

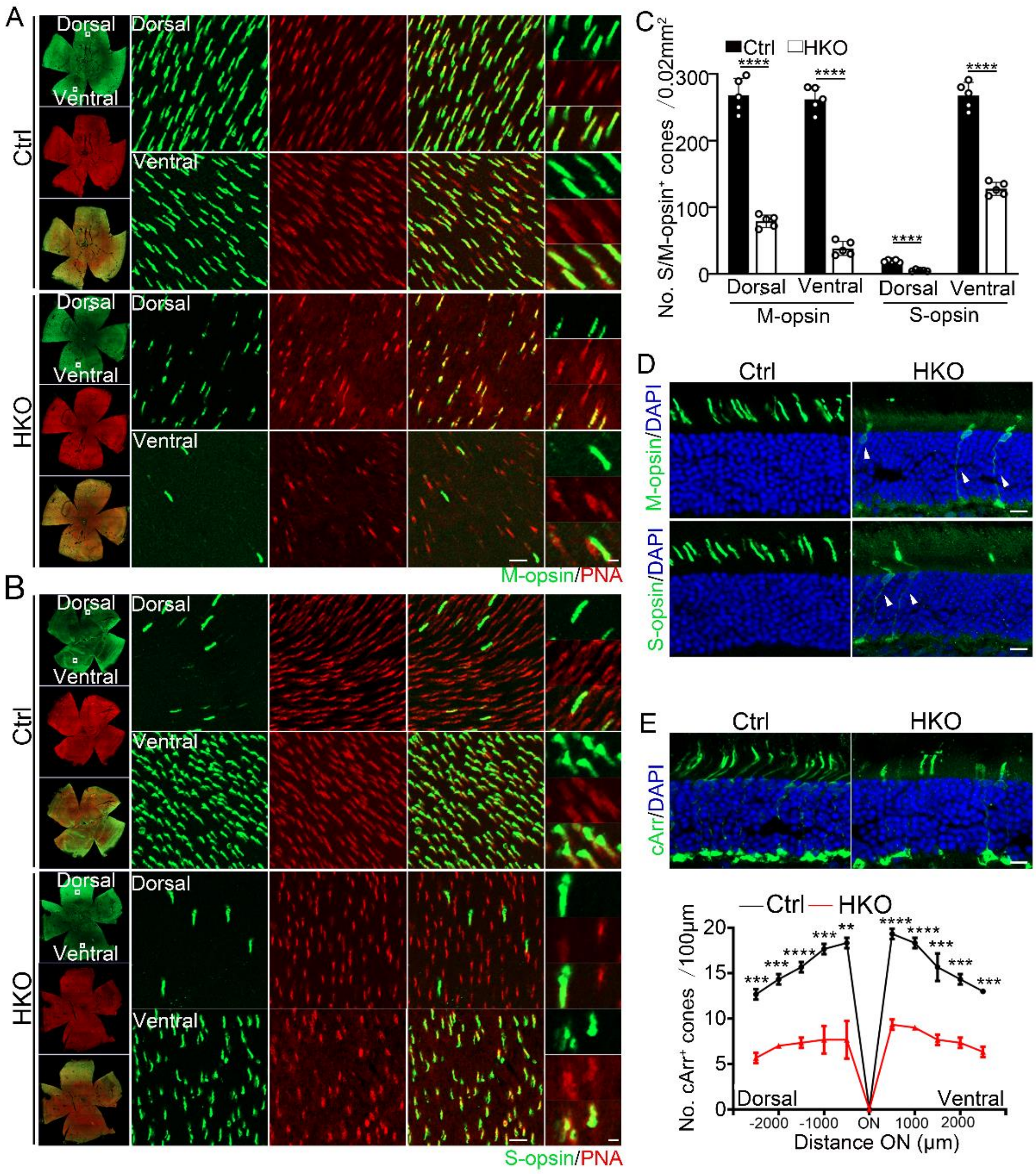

Supplemental Figure 7. Cone cells underwent a progressive loss in HKO mice. (**A** and **B**) Immunostaining of retina flat-mount from 16-week-old Ctrl and HKO mice with M-opsin/ S-opsin (greed) and PNA (red). Scale bars, 50 μm. Representative images from the dorsal and ventral retinal quadrant are shown. Scale bars, 20 μm. Inset images showed a cropped and zoomed image. Scale bars, 5 μm. (**C**) Quantification of the number of M-opsin- and S-opsin-positive cones at both dorsal and ventral side of the retain per field (Student's *t* test, n=5). (**D**) Retinal cryosections from 16-week-old Ctrl and HKO mice were labeled with S-opsin and M-opsin. White arrowheads White

arrowhead indicate the mislocalized M/S-opsin-positive cones. scale bars, 20 μm. (**E**) Representative immunofluorescence images of cArr (green) of retina sections from 16-week-old Ctrl and HKO mice and the number of cArr-positive cones per 500 μm field was quantified. Scale bars, 25 μm. Data are presented as the mean ± SD. Analyzed by multiple 2-tailed *t* tests with the Holm-Sidak method to correct for multiple comparisons. **p < 0.01; ***p < 0.001; ****p < 0.0001.

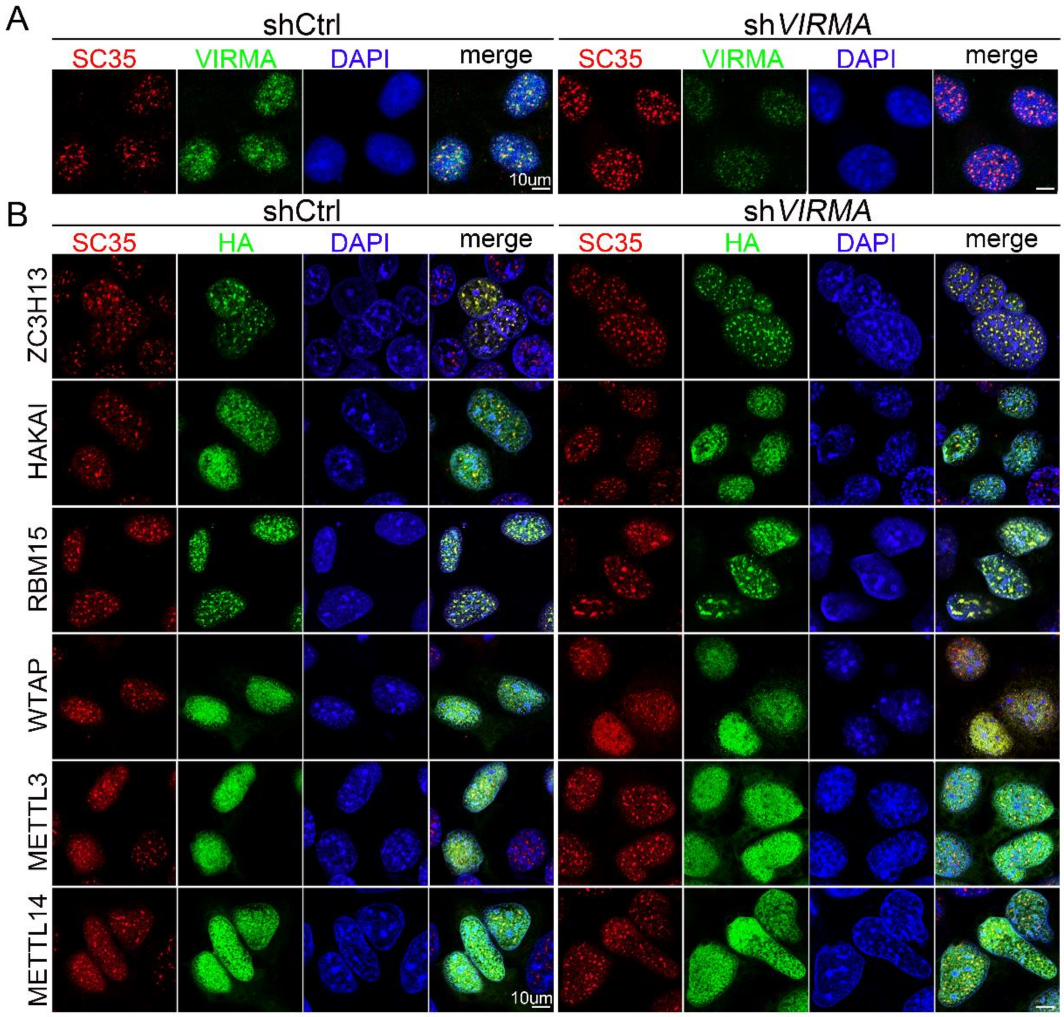

Supplemental Figure 8. Subcellular localization of writers in loss of VIRMA. (**A**) Immunofluorescence staining of VIRMA (green), SC35 (red) and DAPI (blue, cell

nuclei) in *VIRMA* knockdown and control 293STF cells. Scale bar, 10 µm. (**B**) Immunofluorescence analysis of ZC3H13 (green), HAKAI (green), RBM15 (green), WTAP (green), METTL3 (green), METTL14 (green), SC35 (red), and DAPI (blue, cell nuclei) in *VIRMA* knockdown and control 293STF cells. Scale bar, 10 µm.

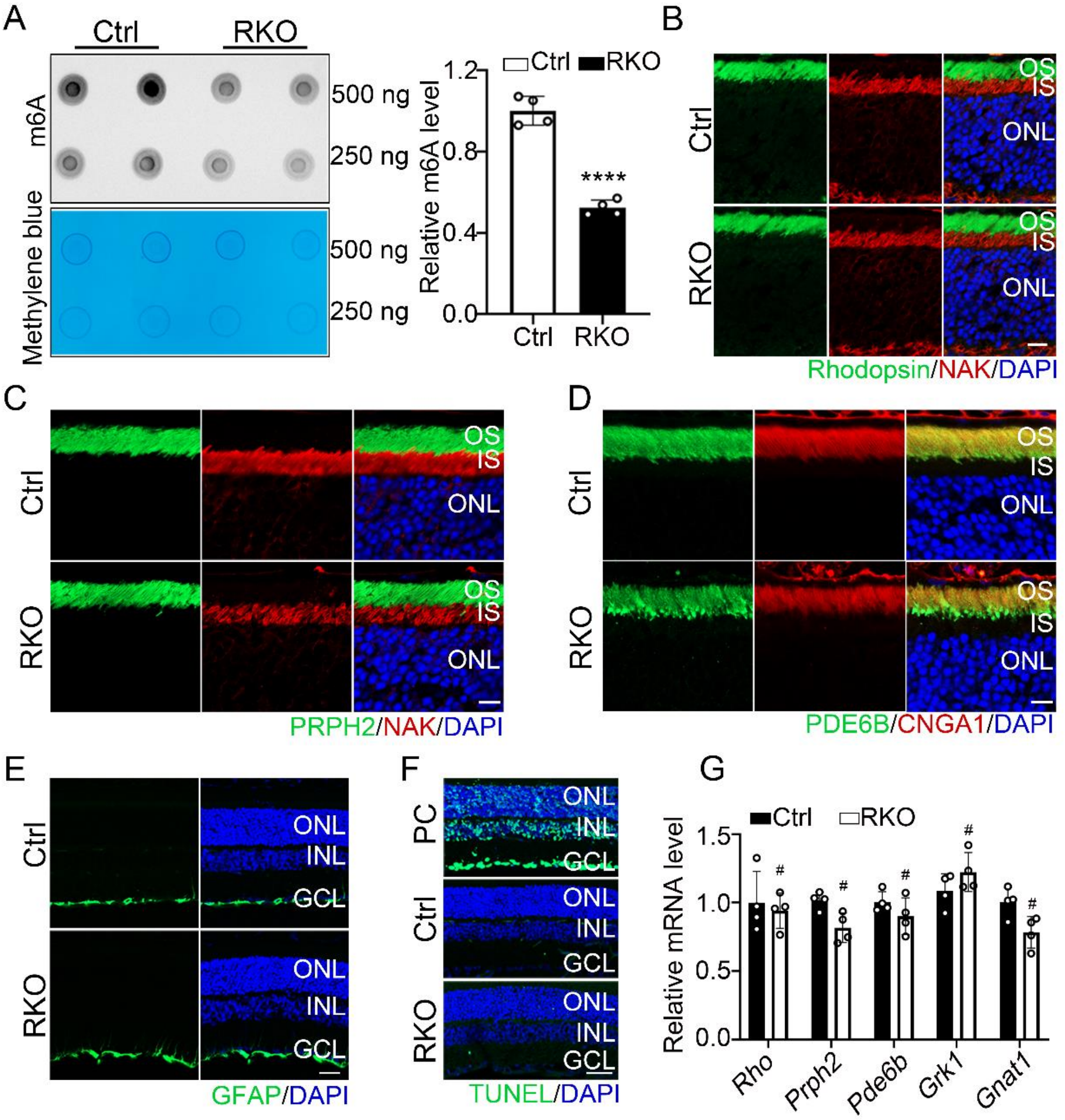

Supplemental Figure 9. 3-week-old RKO mice showed normal retinal structure. (**A**) m^{6}A dot blot assay of global m^{6}A abundance in retinas from 3-week-old Ctrl and RKO mice. Methylene blue staining was used as a loading control (Student's *t* test, n=4).

(**B**-**D**) Retinal cryosections from 3-week-old mice were stained with OS marker Rhodopsin, PRPH2, PDE6B, CNGA1, and IS marker NaK ATPase. Scale bars, 25 µm. (**E**) Retina cryosections from 3-week-old mice were stained with GFAP (green). Scale bars, 20 µm. (**F**) Immunofluorescence labelling of retina cryosections from 3-week-old mice with the TUNEL assay kit. DNase I-treated sections served as the positive control (PC). Scale bars, 20 µm. (**G**) RT-qPCR verified the mRNA expression of visual perception gene from Ctrl and RKO mice. (Student's *t* test, n=4). OS, outer segment; IS, inner segment; ONL, outer nuclear layer; INL, inner nuclear layer; GCL, ganglion cell layer. Data are presented as the mean ± SD. ****$p < 0.0001$; #, no significant difference.

Supplementary Table 2. List of the PCR sets primers used genotyping in this study.

Name	Sequence
Virma-loxp-F	ATCTCTCTGTATGCCACATAGATGC
Virma-loxp-R	GATGATGGAACTGGAGAGAAGTC
Srsf3-loxp-F	TGAAGCTGTAGGATAGGCCATTTG
srsf3-loxp-R	TTGTCCTCTCTTCATTTGTCCAGA
RHO-Cre-F	TCAGTGCCTGGAGTTGCGCTGTGG
RHO-Cre-R	CTTAAAGGCCAGGGCCTGCTTGGC
HRGP-Cre-F	GAACGCACTGATTTCGACCA
HRGP-Cre-R	GCTAACCAGCGTTTTCGTTC
CAG-Virma-P1	TCAGATTCTTTTATAGGGGACACA
CAG-Virma-P2	TAAAGGCCACTCAATGCTCACTAA
CAG-Virma-P3	ATGGCGGTGGACTCGTCTATG
CAG-Virma-P4	AGGAGAAATGGGCTCAAAGTAATC
ROSA26-tdTomato-F	CACTTGCTCTCCCAAAGTCG
ROSA26-tdTomato-R	TAGTCTAACTCGCGACACTG
ROSA26-tdTomato-KI	GTTATGTAACGCGGAACTCC

Supplementary Table 3. List of the antibodies used in this study.

Name	Company	Catalog	Usage
Anti-Rhodopsin (Rabbit polyclonal)	Cell Signaling Technology	D4B9B	IHC(1:200)
Anti-PRPH2 (Rabbit polyclonal)	Proteintech	18109-1-AP	IHC(1:200);WB(1:2000)
Anti-GRK1 (Rabbit polyclonal)	Proteintech	24606-1-ap	IHC(1:200);WB(1:2000)
Anti-PDE6B (Rabbit polyclonal)	Proteintech	22063-1-AP	IHC(1:200)
Anti-CNGA1 (Mouse polyclonal)	Abcam	ab253296	IHC(1:200)
594-conjugated PNA	Vector Laboratories	RL1072	IHC(1:200)
Anti-M-Opsin (Rabbit polyclonal)	Millipore	AB5405	IHC(1:200);WB(1:2000)
Anti-S-Opsin (Rabbit polyclonal)	Millipore	AB5407	IHC(1:200);WB(1:2000)
Anti-Cone Arrestin (Rabbit polyclonal)	Sigma Aldrich	AB15282	IHC(1:200)
Anti-GFAP (Rabbit polyclonal)	Cell Signaling Technology	e4l7m	IHC(1:200);WB(1:2000)
Anti-m^6A (Rabbit polyclonal)	Cell Signaling Technology	D9D9W	IHC(1:200)
Anti-SC35 (Mouse polyclonal)	Abcam	ab11826	IHC(1:200)
Anti-KIAA1429 (Rabbit polyclonal)	Abcam	ab71136	IHC(1:200);WB(1:2000)
Anti-HA (Rabbit polyclonal)	Abmart	M20003	IHC(1:200)
Anti-HSP60 (Rabbit polyclonal)	Proteintech	15282-1-AP	IHC(1:200)
Anti-SRSF3 (Rabbit polyclonal)	Abcam	ab198291	IHC(1:200);WB(1:2000)
Anti-AC-Tublin (Mouse polyclonal)	Sigma Aldrich	T7451-25UL	IHC(1:200)
Anti-CEP164 (Rabbit polyclonal)	Proteintech	22227-1-AP	IHC(1:200)
Anti-IBA1 (Rabbit polyclonal)	Wako	019-19741	IHC(1:200);WB(1:2000)
Anti-METTL3 (Rabbit polyclonal)	Abcam	ab195352	WB(1:2000)
Anti-METTL14 (Rabbit polyclonal)	Sigma	SAB5700855	WB(1:2000)

Anti-WTAP (Rabbit polyclonal)	Proteintech	10200-1-AP	WB(1:2000)
Anti-RBM15 (Rabbit polyclonal)	Proteintech	10587-1-AP	WB(1:2000)
Anti-CBLL1 (Rabbit polyclonal)	Proteintech	21179-1-AP	WB(1:2000)
Anti-β-Actin (Rabbit polyclonal)	Proteintech	20536-1-AP	WB(1:2000)
Anti-Rhodopsin (Rabbit polyclonal)	Cell Signaling Technology	D1N7X	WB(1:2000)
Anti-GNAT1 (Rabbit polyclonal)	Proteintech	55167-1-AP	WB(1:2000)
Anti-RDH12 (Rabbit polyclonal)	Proteintech	13289-3-AP	WB(1:2000)
Anti-POLG2 (Rabbit polyclonal)	Abclonal	A6695	WB(1:2000)
Anti-RGS9 (Rabbit polyclonal)	Proteintech	17970-1-AP	WB(1:2000)
Anti-PDE6G (Rabbit polyclonal)	Proteintech	18151-1-AP	WB(1:2000)
Anti-CRE (Rabbit polyclonal)	Cell Signaling Technology	15036T	IHC(1:200)
HRP-conjugated Goat Anti-Mouse IgG	Proteintech	SA00001-1	WB(1:5000)
HRP-conjugated Goat Anti-Rabbit IgG	Proteintech	SA00001-2	WB(1:5000)
Goat Anti-Mouse IgG 488	Thermo Fisher Scientific	A11029	IHC(1:500)
Goat Anti-Mouse IgG 594	Thermo Fisher Scientific	A-11005	IHC(1:500)
Goat Anti-Rabbit IgG 488	Thermo Fisher Scientific	A-11008	IHC(1:500)
Goat Anti-Rabbit IgG 594	Thermo Fisher Scientific	A-11012	IHC(1:500)
DAPI	Biolegend	422801	IHC(1:1000)

Supplementary Table 4. List of the RT-qPCR primers used in this study.

Gene name	Forward (5’-3’)	Reverse (5’-3’)
Bcl2a	TTTGAGTTCGGTGGGGTCAT	CTGGGGCCATATAGTTCCACAA
Sod1	GGAACCATCCACTTCGAGCA	CCCATGCTGGCCTTCAGTTA
Sod2	GCCTGCTCTAATCAGGACCC	GGTAGTAAGCGTGCTCCCAC

Virma	ATGTCATGGAAACTGCACCTC	GAGTGCTGAAAACCAAACCCA
Gnat1	GACTCCAGGATATGTGCCCA	CACCAGCACCATGTCGTAAG
Guca1b	AAGCGCTTCTTCAAGGTCAC	GCTTGTAAATCGCCTCCACA
Pde6g	AAGGCAGTTCAAGAGCAAGC	GAGGGGTCTTCGCGGTT
Rho	CACCACCACCCTCTACACAT	GATAGCGTGATTCTCCCCGA
Slc24a1	AGATGGTGGAGGGGAAAGTG	GGAGGAAGAGGTAGATGGCC
Mdm1	CCCAGAGCATAGATCCCAGG	TTGGATGCAAACACTGGAGC
Prph2	CCTGGCTTACGGACTCAAGA	ATCCACGTTGCTCTTGATGC
Rdh12	GTTGGAGAGGCTGAAGGAGT	CTCTGACAATACAACGCCCG
Rgs9	AGATGCGAGTGGAGAGATGG	TTGGACTGATCGCCGTACTT
Rs1	CAGAGGATGAGGGTGAGGAC	GCCTTGTTTGCTGTCCATGA
Rgs9bp	AATGAAAGTCAACGTGCCCC	GCTCAGCTTTGCCACACATA
Cc2d2a	ATTCAACTGGCCAGAGAGCT	CCCACTTCCAACTCCCTCAT
Pde6b	CCCCTGACTCTGAGATCGTC	TGATCACAGCCACGACATCT
β-actin	GGCTGTATTCCCCTCCATCG	CCAGTTGGTAACAATGCCATGT
