# Supplementary material for: VIRMA modulates function of photoreceptor cells through m6A modification and alternative splicing
Source: JCI Insight. 2026 Mar 19;11(9):e197880. doi: 10.1172/jci.insight.197880 (PMC13232022; doi:10.1172/jci.insight.197880)

Figure 4C

VIRMA (202kDa)

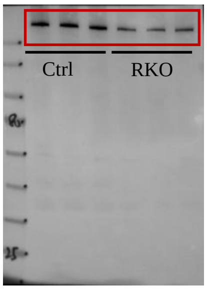

METTL3 (64kDa)

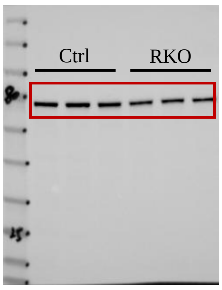

METTL14 (60kDa)

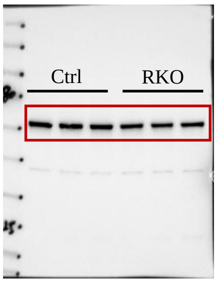

WTAP (50kDa)

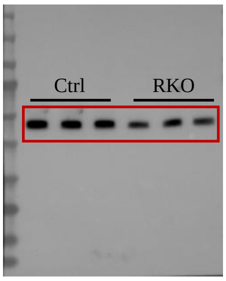

RBM15 (100kDa)

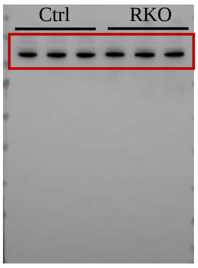

HAKAI (60kDa)

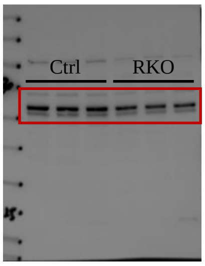

β-Actin (42kDa)

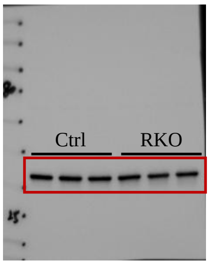

Figure 5F

RHO (39,75,150kDa)

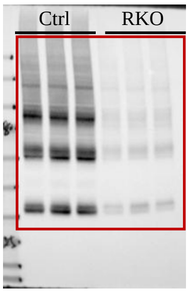

GNAT1 (40kDa)

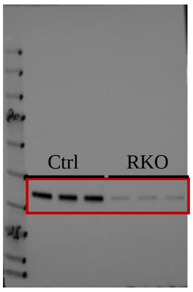

PDE6B (100kDa)

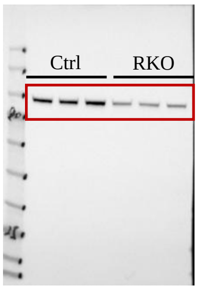

PRPH2 (30kDa)

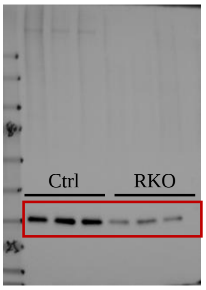

RDH12 (35kDa)

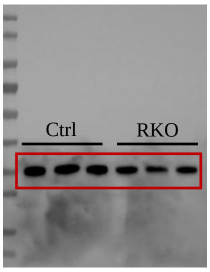

β-Actin(42kDa)

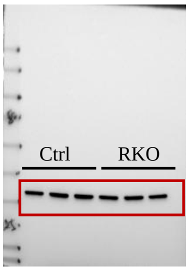

Figure 6A

Input

VIRMA (202kDa) SRSF3 (20kDa)

Flag-IP

VIRMA SRSF3

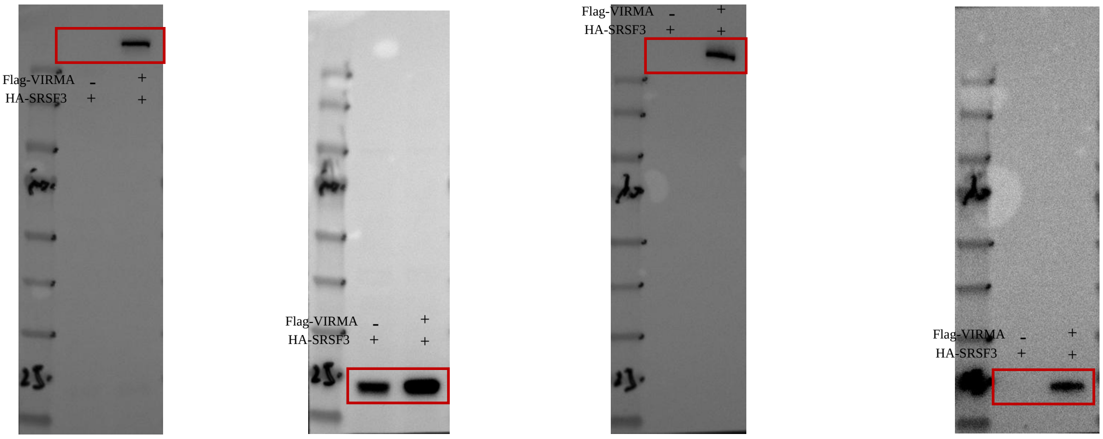

Figure 6D

Impdh1

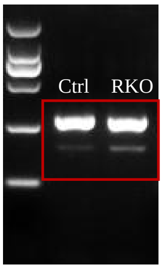

Figure 6G

Polg2

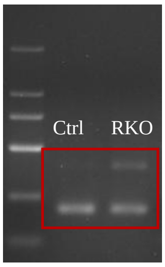

Figure 6I

POLG (55kDa) β-Actin(42kDa)

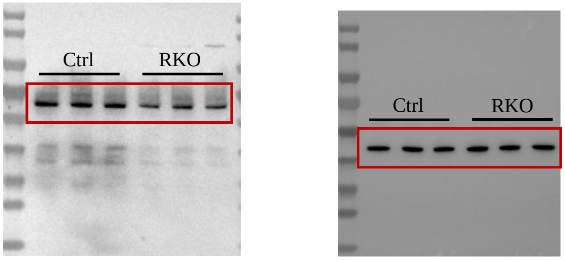

Figure 7H

SRSF3(20kDa) RHO (39,75,150kDa) GNAT1(40kDa) GRK1 (64kDa)

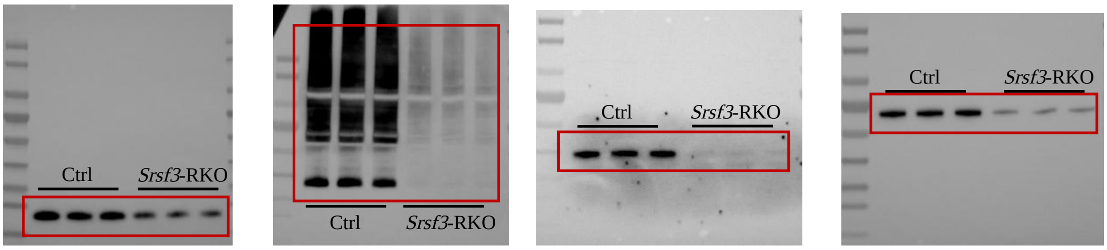

PDE6A(100kDa) PDE6B(100kDa) PRPH2 (30kDa) β-Actin (42kDa)

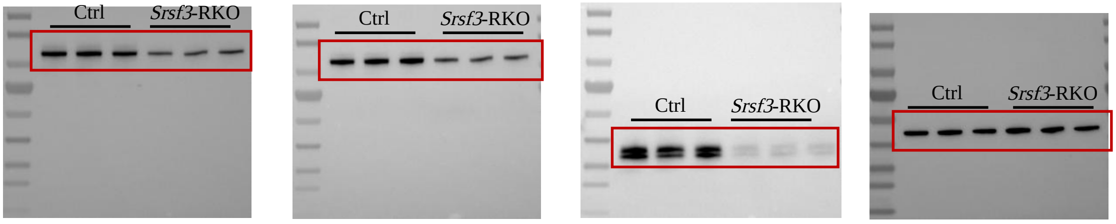

Figure 8E

Flag(202kDa)

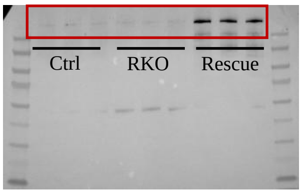

RHO(39kDa)

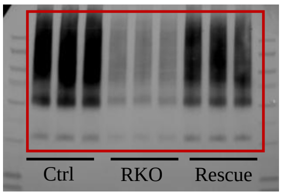

GNAT1(40kDa)

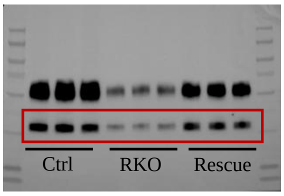

PDE6B(100kDa)

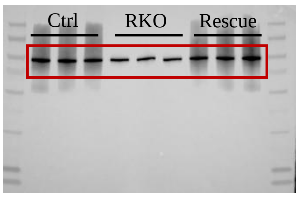

PRPH2 (30kDa)

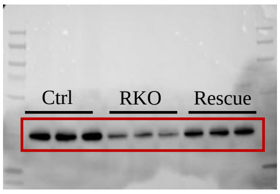

RGS9 (60kDa)

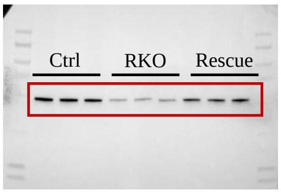

PDE6G(13kDa)

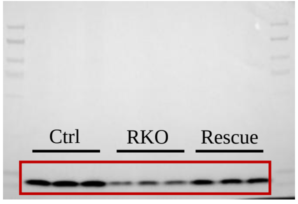

RDH12 (35kDa)

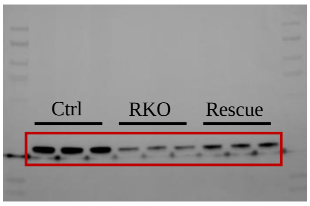

β-Actin (42kDa)

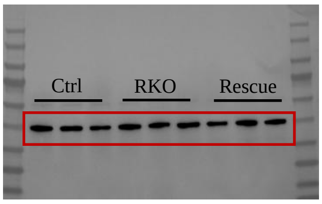

Supplementary Figure 1B

Virma-flox

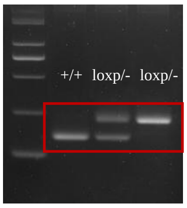

RHO-Cre

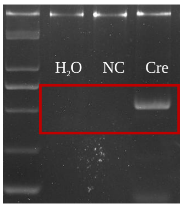

HRGP-Cre

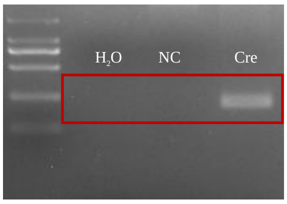

Supplementary Figure 1E

VIRMA(202kDa)

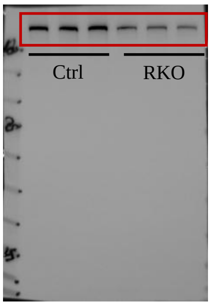

β-Actin(42kDa)

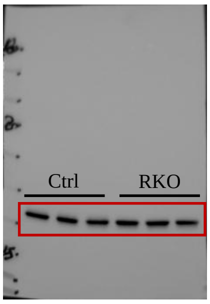

Supplementary Figure 4C

GFAP(50kDa) IBA1(17kDa) β-Actin(42kDa)

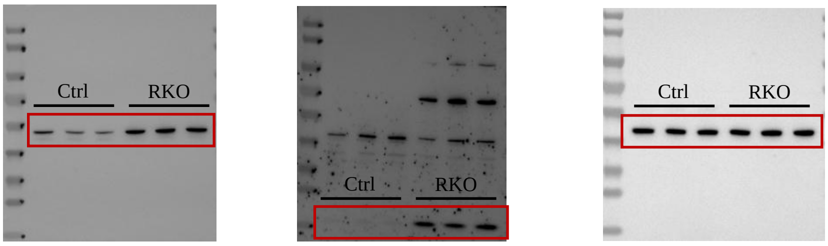

Supplementary Figure 5D

S-opsin(43kDa) M-opsin(75kDa) ARR3(50kDa) β-Actin(42kDa)

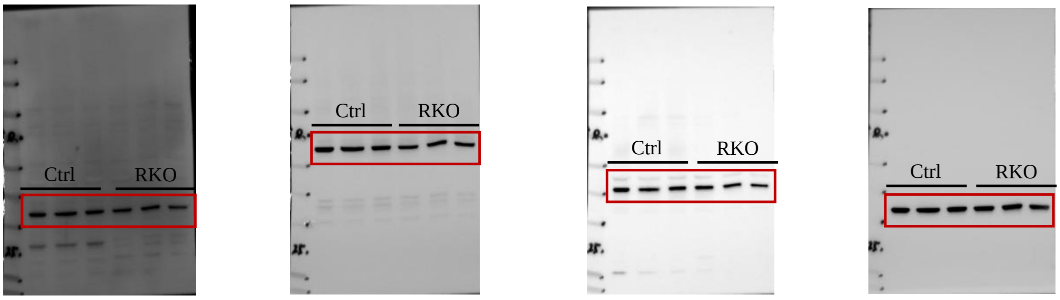

Supplement: Unedited blot and gel images [file jciinsight-11-197880-s188.pdf]
